# Chelator PBT2 Forms a Ternary Cu^2+^ Complex with β-Amyloid That Has High Stability but Low Specificity

**DOI:** 10.3390/ijms24119267

**Published:** 2023-05-25

**Authors:** Simon C. Drew

**Affiliations:** 1 Brain–Immune Communication Lab, Institut Pasteur, Université Paris Cité, F-75015 Paris, France; sdrew@pasteur.fr or sdrew@unimelb.edu.au; 2 Department of Medicine (Royal Melbourne Hospital), The University of Melbourne, Victoria 3010, Australia

**Keywords:** 8-hydroxyquinoline, PBT2, amyloid, copper, ternary, terdentate, antimicrobial

## Abstract

The metal chelator PBT2 (5,7-dichloro-2-[(dimethylamino)methyl]-8-hydroxyquinoline) acts as a terdentate ligand capable of forming binary and ternary Cu^2+^ complexes. It was clinically trialed as an Alzheimer’s disease (AD) therapy but failed to progress beyond phase II. The β-amyloid (Aβ) peptide associated with AD was recently concluded to form a unique Cu(Aβ) complex that is inaccessible to PBT2. Herein, it is shown that the species ascribed to this binary Cu(Aβ) complex in fact corresponds to ternary Cu(PBT2)N_Im_^Aβ^ complexes formed by the anchoring of Cu(PBT2) on imine nitrogen (N_Im_) donors of His side chains. The primary site of ternary complex formation is His6, with a conditional stepwise formation constant at pH 7.4 (Kc [M^−1^]) of logKc = 6.4 ± 0.1, and a second site is supplied by His13 or His14 (logKc = 4.4 ± 0.1). The stability of Cu(PBT2)N_Im_^H13/14^ is comparable with that of the simplest Cu(PBT2)N_Im_ complexes involving the N_Im_ coordination of free imidazole (logKc = 4.22 ± 0.09) and histamine (logKc = 4.00 ± 0.05). The 100-fold larger formation constant for Cu(PBT2)N_Im_^H6^ indicates that outer-sphere ligand–peptide interactions strongly stabilize its structure. Despite the relatively high stability of Cu(PBT2)N_Im_^H6^, PBT2 is a promiscuous chelator capable of forming a ternary Cu(PBT2)N_Im_ complex with any ligand containing an N_Im_ donor. These ligands include histamine, L-His, and ubiquitous His side chains of peptides and proteins in the extracellular milieu, whose combined effect should outweigh that of a single Cu(PBT2)N_Im_^H6^ complex regardless of its stability. We therefore conclude that PBT2 is capable of accessing Cu(Aβ) complexes with high stability but low specificity. The results have implications for future AD therapeutic strategies and understanding the role of PBT2 in the bulk transport of transition metal ions. Given the repurposing of PBT2 as a drug for breaking antibiotic resistance, ternary Cu(PBT2)N_Im_ and analogous Zn(PBT2)N_Im_ complexes may be relevant to its antimicrobial properties.

## 1. Introduction

The compound 5,7-dichloro-2-[(dimethylamino)methyl]-8-hydroxyquinoline (PBT2) is a terdentate Cu^2+^ and Zn^2+^ chelator that was previously trialed as a therapeutic to treat Alzheimer’s disease (AD). Its anticipated mechanism of action was based on the controversial “metals hypothesis”, which proposed that AD results from aberrant interactions of the β-amyloid (Aβ) peptide with endogenous transition metal ions, notably Cu^2+^, causing Aβ to misfold and generate reactive oxygen species (ROS) [1,2]. PBT2 was proposed to prevent these interactions, sequester transition metal ions thought to be trapped within extracellular β-amyloid aggregates, and then enable cellular uptake of these ions by ionophore action [3]. However, repeated phase II clinical trials ultimately provided no evidence for cognitive efficacy of PBT2 in patients with prodromal or mild AD [4,5].

**Figure 1:**
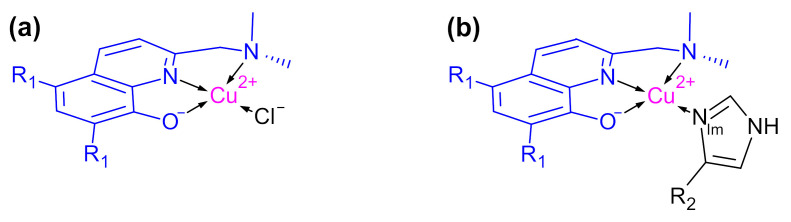
Structure of (**a**) binary and (**b**) ternary Cu^2+^ complexes of PBT2 (R_1_ = Cl) and its non-chlorinated homologue (R_1_ = H). The imine nitrogen (N_Im_) is supplied by ligands including imidazole (R_2_ = H), histamine (R_2_ = CH_2_CH_2_NH_3_^+^), L-His (R_2_ = CH_2_(COO^−^)CHNH_3_^+^), and His side chains (R_2_ = protein).

Using electron paramagnetic resonance (EPR) spectroscopy, the Cu^2+^ coordination of this class of ligand (L) was first characterized using the non-chlorinated homologue of PBT2 (Figure 1), which was shown to form a terdentate CuL complex and a five-coordinate CuL_2_ complex [6]. The proposed structure of CuL_2_ has been replicated in the crystal structure of PBT2 [7], and the UV–vis and EPR spectroscopic properties of Cu(PBT2) and Cu(PBT2)_2_ have been shown to mirror those of the non-chlorinated homologue [6,7,8,9] (Table S1). Early EPR studies also showed that both ligands form ternary CuLN_Im_^X^ complexes (Figure 1) in which the labile Cl^−^ ligand of CuL is replaced with an imine (N_Im_) donor ligand provided by X = imidazole, histamine, L-His, or proteins such as α-synuclein and Aβ (see, in particular, Figure S33 of ref. [6]). However, despite recently identifying a similar EPR spectroscopic signature using a Cu/PBT2/Aβ_1–42_ mixture, George and co-workers ascribed the signal to a unique PBT2-inaccessible Cu(Aβ) species and concluded that there was no evidence to support formation of a ternary Cu^2+^ complex [10]. Since this could be interpreted by some as a reason for the failure of PBT2 in AD clinical trials, it is important to resolve this contradiction.

To address the above issue, we used EPR spectroscopy to analyze the species distributions in Cu/PBT2/Aβ mixtures in unprecedented detail and identify the number and stabilities of ternary Cu(PBT2)N_Im_^X^ complexes formed with X = Aβ_1–40_. 

## 2. Results

**Figure 2:**
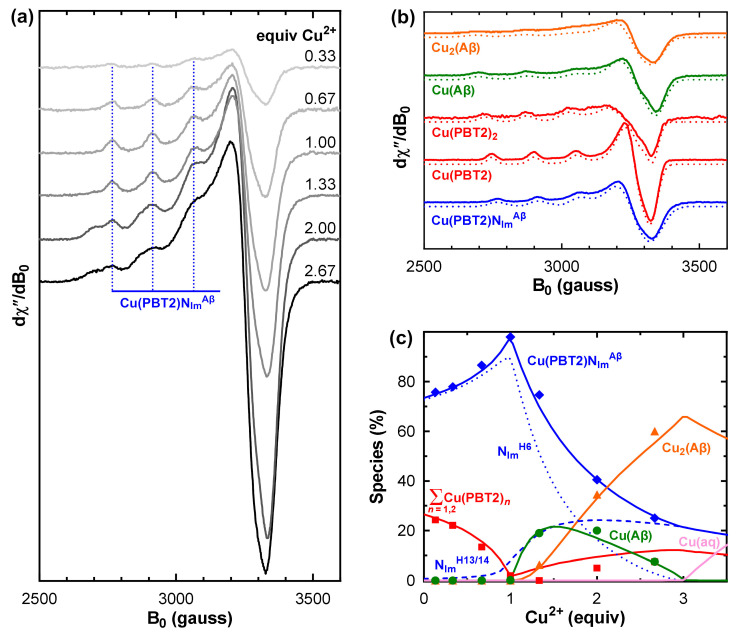
Determination of the number and stabilities of ternary Cu(PBT2)(Aβ_1–40_) interactions. (**a**) EPR spectra of ^65^Cu/PBT2/Aβ_1–40_ *n*:1:1 (*n* = 0.1–2.67) in PBS pH 7.4 (0.2 mM PBT2/Aβ_1–40_). The group of vertical lines indicates the ^65^Cu hyperfine splitting (*A_z_*) associated with Cu(PBT2)N_Im_^Aβ^. (**b**) Normalized basis set (solid lines) used for the decomposition of the spectra in panel *a* (see Figures S1–S3 for details). Dotted lines show spectra simulated using the parameters in Table 1. The Cu_2_(Aβ_1–40_) simulation is a weighted summation of spectra obtained using “first site” parameters (50%) and “second site” parameters (50%). Vertical scales in panels *a* and *b* are different. (**c**) Experimental species distributions (points) resulting from normalization and decomposition of the spectra in panel *a* (Figure S4), and theoretical distributions (solid lines) calculated using the relevant formation constants in Table 2 (see also Figure S5 for a comparison of relative and absolute Cu^2+^ speciation). The solid blue line in panel *c* shows the sum of Cu(PBT2)N_Im_^H6^ (dotted line) and Cu(PBT2)N_Im_^H13/14^ (dashed line). Cu(aq) denotes aqueous Cu^2+^ (pink). Experimental conditions: temperature, 77 K; microwave power, 10 mW; microwave frequency, 9.425 GHz; modulation amplitude, 8 G; sweep time, 84 s; time constant, 82 ms; averages, 4.

To characterize the Cu^2+^ complexes formed by PBT2 in the presence of Aβ, we titrated an equimolar mixture of PBT2 and Aβ_1–40_ in PBS pH 7.4 with Cu^2+^ and acquired the corresponding EPR spectra (Figure 2a). To determine the species distribution, each spectrum was decomposed into a linear superposition of basis spectra (Figure 2b) corresponding to Cu(PBT2), Cu(PBT2)_2_, Cu(Aβ_1–40_), Cu_2_(Aβ_1–40_), and the putative Cu(PBT2)N_Im_^Aβ^ complex. The spectrum of Cu(PBT2) was obtained at equimolar Cu/PBT2 stoichiometry and that of Cu(PBT2)_2_ at sub-stoichiometric ratios (Figure S1). The spectra of Cu(Aβ) and Cu_2_(Aβ) were acquired at Cu/Aβ ratios of 1:1 and 2.5:1 (Figure S2). Attempts to use linear combinations of normalized Cu(PBT2), Cu(PBT2)_2_, Cu(Aβ), and Cu_2_(Aβ) spectra failed to reproduce the EPR spectra of Cu/PBT2/Aβ_1–40_ *n*:1:1 (*n* = 0–2.67), indicating that additional species must be formed. Indeed, the dominant spectral features did not resemble any of those of the above four species (Figure 2b and Figure S3). Rather, they corresponded closely to those of previously characterized CuLN_Im_ complexes (Table 1) [6,8,11], indicating that Cu(PBT2) can anchor on the imine nitrogen (N_Im_) of the His side chains of Aβ_1–40_. 

To quantify the number and stabilities of ternary Cu(PBT2)N_Im_^Aβ^ interactions, it is important to consider how the His residues participate in binary Cu(Aβ) complexes. At pH 7.4, Cu(Aβ) is dominated by {NH_2_^D1^, C=O^D1^, N_Im_^H6^, N_Im_^H13^} and {NH_2_^D1^, C=O^D1^, N_Im_^H6^, N_Im_^H14^} coordination spheres with indistinguishable EPR spectra [12,13,14]. Thus, His6 is absolutely required to form Cu(Aβ), whereas only one of His13 or His14 is needed. Thus, anchoring of Cu(PBT2) on His6 to form Cu(PBT2)N_Im_^H6^ will occur at the expense of Cu(Aβ), whereas anchoring on one of His13 or His14 to form Cu(PBT2)N_Im_^H13/14^ will not. Sequential binding of a second Cu^2+^ ion by Cu(Aβ) generates Cu_2_(Aβ). The coordination in Cu_2_(Aβ) remains poorly defined, with one suggestion that binding of the second Cu^2+^ ion changes the coordination of the first [15]. However, it will be shown below that a satisfactory explanation of the species distributions requires that His6 remains Cu^2+^-bound and His13 or His14 non-coordinated in Cu_2_(Aβ).

The conditional constants (Kc) for formation of Cu(PBT2), Cu(PBT2)_2_, Cu(Aβ), and Cu_2_(Aβ) at pH 7.4 have all been previously determined (Table S2), which greatly simplified the task of determining the species distribution of the Cu/PBT2/Aβ_1–40_ *n*:1:1 system. Using these values and the EPR basis spectra (Figure 2b), we fitted the series of EPR spectra in Figure 2a as a function of the unknown constants KCuLNImH6CuLc and KCuLNImH13/14CuLc (see Section 4.3 for detail). The best agreement between the experimental and theoretical species distributions (Figure 2c) was obtained for logKCuLNImH6CuLc = 6.4 ± 0.1 and logKCuLNImH13/14CuLc = 4.4 ± 0.1 (Table 2). Allowing the conditional formation constants KCu(Aβ)Cuc and KCu2(Aβ)Cu(Aβ)c to vary beyond their generally accepted ranges (Table S2) worsened the fit. Although we did not refine the value of KCuL2CuLc, we found that large changes from its published value (Table 2) also worsened the fit unless KCu(Aβ)Cuc was set to a value beyond its accepted range.

The general appearance of the species distributions for Cu/PBT2/Aβ_1–40_ *n*:1:1 (Figure 2c and Figure S5) can be understood as follows: For small *n*, the 1000-fold greater stability of Cu(PBT2) compared with Cu(Aβ) (Table 2) ensures that Cu^2+^ will first bind to PBT2, with the identity of the fourth in-plane ligand then being determined by the relative magnitudes of the stepwise constants KCuLNImH6CuLc>KCuL2CuLc≫KCuLNImH13/14CuLc. Thus, for *n* < 1, Cu^2+^ is predominantly bound in a Cu(PBT2)N_Im_^H6^ complex with a minor quantity of Cu(PBT2)_2_. For *n* = 1, Cu(PBT2)_2_ is almost entirely replaced by Cu(PBT2)N_Im_^H6^ and Cu(PBT2)N_Im_^H13/14^, which only require one PBT2 molecule per Cu^2+^ ion. For *n* > 1, with no free PBT2 molecules available, the additional Cu^2+^ is coordinated in the next most stable binary complex, which is Cu(Aβ) (logKCu(Aβ)Cuc = 10.0 ± 0.1). However, because Cu(Aβ) coordination requires His6, some Cu(PBT2) detaches from His6 and anchors instead on a His13 or His14 side chain, albeit with lower stability, to form Cu(PBT2)N_Im_^H13/14^. As the available sites at His6 are gradually filled by Cu(Aβ), stepwise addition of a second Cu^2+^ ion to the peptide occurs (logKCu2(Aβ)Cu(Aβ)c = 8.0 ± 0.1) to form Cu_2_(Aβ). Maximum occupancy of the Cu/PBT2/Aβ_1–40_ *n*:1:1 system is reached at *n* = 3, with two Cu^2+^ ions bound to the “first” and “second” sites of Aβ, and a third Cu^2+^ ion bound either to Cu(PBT2) that is free (minor) or anchored to His13/14 of Cu_2_(Aβ) in a Cu(PBT2)N_Im_^H13/14^ complex (major).

More than three Cu^2+^ ions cannot be accommodated by an equimolar PBT2/Aβ mixture, with the excess Cu^2+^ ions existing as aqueous copper that will precipitate as [Cu(OH)_2_]*_n_* at pH 7.4. Inclusion of Cu(PBT2)N_Im_^H13/14^, whose formation does not depend on the Cu^2+^ loading state of Aβ, was essential to fit the experimental data. Alternative explanations of the physical origin of the lower-affinity ternary complex, such as a change in coordination of the first-bound Cu^2+^ ion in Cu_2_(Aβ), can be ruled out because the concentration of Cu_2_(Aβ) relative to that of the ternary complex is too low at *n* < 2 (Figure S5).

The fact that KCuLNImH6CuLc is 100-fold larger than KCuLNImH13/14CuLc indicates that either Cu(PBT2)N_Im_^H6^ is stabilized by favorable outer-sphere ligand–peptide interactions and/or that Cu(PBT2)N_Im_^H13/14^ is destabilized by such interactions. To distinguish between these possibilities, we repeated the EPR analyses using Cu/PBT2/X 1:1:*n* systems with relatively unstructured N_Im_ donor ligands from X = imidazole (Figures S6–S8) and histamine (Figures S9–S11). The EPR spectra of the isolated ternary Cu(PBT2)N_Im_^X^ complexes were characterized by the same parameters as Cu(PBT2)N_Im_^Aβ^ spectra (Table 1), confirming that each of these ternary Cu^2+^ complexes involves monodentate N_Im_ coordination of the co-ligand (Figure 1). The difference between KCuLNImimidazoleCuLc and KCuLNImH13/14CuLc was within experimental error, whereas KCuLNImhistamineCuLc was slightly lower than these constants (Table 2). Thus, we may conclude that the stability of Cu(PBT2)N_Im_^H13/14^ is not strongly influenced by outer-sphere ligand–peptide interactions, whereas such interactions greatly enhance the stability of Cu(PBT2)N_Im_^H6^.

To independently verify the EPR method for deriving the conditional formation constants, we also determined the stability of the ternary Cu^2+^ complex of the non-chlorinated homologue of PBT2 (L′) with imidazole (Figures S12–S14) and compared the value with that previously determined using potentiometric titrations [11]. After pH correction of the absolute stability constants (Table S3),KCuL′NImimidazoleCuL′c was not significantly different from that determined here using EPR (Table S2) and, similar to PBT2, slightly higher than KCuL′NImhistamineCuL′c (Figures S15–S17).

## 3. Discussion

EPR spectroscopy isolated a common Cu(PBT2)N_Im_^Aβ^ spectrum (Figure S3) for both Cu(PBT2)N_Im_^H6^ and Cu(PBT2)N_Im_^H13/14^ because they have very similar first coordination spheres. Nevertheless, as has been shown for other terdentate Cu^2+^ chelators [16,17], ternary complexes with different N_Im_ donor ligands can be distinguished based on their distinct formation constants, which are determined by outer-sphere interactions to which continuous-wave EPR is typically insensitive. Importantly, the spectroscopic signature of Cu(PBT2)N_Im_^Aβ^ isolated here for Cu/PBT2/Aβ_1–40_ *n*:1:1 closely matches that reported for the species isolated in Cu/PBT2/Aβ_1–42_ 1:2:1 [10] (Table 1). In the latter study, the authors ascribed this to a unique PBT2-inaccessible Cu(Aβ) complex and concluded that there was no evidence for ternary complex formation. However, it is clear that CuLN_Im_^X^ complexes with this spectroscopic signature are formed by PBT2 with a number of N_Im_ donor ligands X (Table 1 and Table S1). Moreover, we demonstrated the requirement for two such complexes—Cu(PBT2)N_Im_^H6^ and Cu(PBT2)N_Im_^H13/14^—with distinct stabilities (Table 2) to explain the species distributions of Cu/PBT2/Aβ_1–40_ mixtures. The relatively high stability of Cu(PBT2)N_Im_^H6^ compared with complexes formed with other N_Im_ donors might result from stabilizing pi–pi stacking of the aromatic rings of PBT2 and Phe4 or Tyr10, although a combination of electrostatic, steric, and hydrogen-bonding effects may contribute.

Despite the large ternary formation constant for Cu(PBT2)N_Im_^H6^, PBT2 remains a promiscuous Cu^2+^ chelator because it is capable of forming a ternary Cu(PBT2)N_Im_ complex with all N_Im_ donor ligands, including ubiquitous His side chains of peptides and proteins in the biological milieu, whose combined effect should outweigh that of a single Cu(PBT2)N_Im_^Aβ^ complex regardless of its stability. We therefore conclude that PBT2 is capable of accessing Cu(Aβ) complexes with high stability but low specificity. Potential functional implications of Cu(PBT2)N_Im_ complexes have been discussed in our previous studies of the non-chlorinated PBT2 homologue. First, as an alternative to acting as a mobile ion carrier (ionophore) in a lipid membrane, endocytosis of extracellular proteins on which Cu(PBT2) is anchored, followed by release of Cu^2+^ in low-pH and/or reducing intracellular compartments, may contribute to the bulk transport of these ions [6]. Second, their production of ROS in the presence of ascorbate [11], in addition to the modulation of cellular ROS signaling following exposure to this class of ligand [17], contrasts with the originally intended ROS-silencing function of PBT2 [18].

Recently, PBT2 has found renewed interest as an antimicrobial compound. Notably, a number of gram-positive bacteria become re-sensitized to previously resistant classes of antibiotics when these antibiotics are supplemented with PBT2 and Zn^2+^ in mouse models of wound healing [19] and pneumonia [20]. These results have been attributed to multiple bactericidal mechanisms associated with intracellular Zn^2+^ accumulation, including impairment of Mn-dependent superoxide dismutase and production of ROS [21]. Although ligands generally have a greater affinity for Cu^2+^ compared with Zn^2+^ [22], the above effects were observed in response to co-administration of PBT2 (~1 μM) with an excess of Zn^2+^ (~100 μM). Therefore, it remains unclear whether ternary Cu(PBT2)N_Im_ complexes may be formed under these conditions. However, given the ability of PBT2 to also form terdentate Zn^2+^ chelates [7], a contribution from analogous Zn(PBT2)N_Im_ complexes to the antimicrobial activity of Zn/PBT2 may be speculated.

## 4. Materials and Methods

### 4.1. Sample Preparation

Aβ_1–40_ (purity > 95%) was synthesized in the Peptide Technology Facility of the Bio21 Molecular Science and Biotechnology Institute, The University of Melbourne. The lyophilized peptide was dissolved at a nominal concentration of 1 mg/mL in 1,1,1,3,3,3-hexafluoroisopropanol and portioned, then the solvent was allowed to evaporate. The resulting peptide film was resuspended at 4 °C in 20% *v/v* 20 mM NaOH, 70% *v*/*v* ultrapure water (MilliQ; MERCK KGAA, Darmstadt, Germany), and 10% *v/v* 10 × phosphate-buffered saline (PBS; 10 mM phosphate buffer, 2.7 mM KCl, 137 mM NaCl; Sigma). After centrifugation at 14,000× *g* for 15 min at 4 °C, the supernatant was retained and the peptide concentration was immediately determined using ε_214_ = 74,925 M^−1^cm^−1^ [23]. A concentrated stock of ^65^CuCl_2_ was prepared by stirring ^65^CuO (^65^Cu, >99%; Cambridge Isotope Laboratories, Tewksbury, MA, USA) in concentrated HCl, evaporating excess HCl under heat, then adding ultrapure water. PBT2 was synthesized as previously described [24], and a 1 mM stock solution was prepared by solubilizing the hydrochloride salt directly in PBS.

From the above stock solutions, PBT2 and then ^65^CuCl_2_ were added to portions of Aβ_1–40_ to achieve final molar Cu/PBT2/ratios of *n*:1:1 (*n* = 0.33–2.67) with [Aβ_1–40_] = 200 μM. Control samples containing Cu/PBT2 0.5:1, Cu/PBT2 1:1, Cu/Aβ_1–40_ 1:1, and Cu/Aβ_1–40_ 2.5:1 were also prepared. Glycerol (10% *v/v*) was added to the Cu/PBT2 control samples to prevent formation of a concentrated solute phase upon freezing. Immediately after Cu addition, the final solution pH was measured using a micro-probe (Hanna Instruments, Villafranca Padovana, Italy) and adjusted using concentrated NaOH or HCl as required. Samples were transferred to quartz EPR tubes (SQ-707; ATS Life Sciences Wilmad, Vineland, NJ, USA) and snap-frozen in liquid nitrogen within two minutes of the Cu addition.

### 4.2. EPR Spectroscopy

X-band continuous-wave EPR spectra were acquired using a Bruker ESP380E spectrometer fitted with a rectangular TE_102_ microwave cavity and a quartz cold finger insert. Microwave frequencies were measured using an EIP Microwave 548A frequency counter and *g* factors calibrated against the F^+^ line in CaO (*g* = 2.0001 ± 0.0002) [25]. Experimental conditions are indicated in the figure captions. Background correction was performed by subtraction of the buffer-only spectrum.

The “pepper” function in EasySpin v.5.2.33 [26,27] was used to simulate basis spectra using the static Hamiltonian

(1) $$H=\beta_{e}B^{T}\cdot g\cdot S+S^{T}\cdot A\cdot I-g_{n}\beta_{n}B^{T}\cdot I$$

where **S** and **I** are the electron and ^65^Cu nuclear vector spin operators, **g** and **A** are the 3 × 3 electron Zeeman and ^65^Cu electron–nuclear hyperfine coupling matrices, *β*_e_ is the Bohr magneton, *β*_n_ is the nuclear magneton, and **B** is the applied magnetic field vector. Rhombic symmetry or higher was assumed for **g** and **A**, with principal values of *g_x_*, *g_y_*, and *g_z_* and *A_x_*, *A_y_*, and *A_z_*, respectively. The principal values of **g** and **A** and the lineshape parameters (*g*–*A* strain model) were varied iteratively using the “esfit” module in EasySpin to minimize the difference between the experimental and simulated spectra.

### 4.3. Determination of the Ternary Formation Constants for Cu/PBT2/Aβ_1–40_ Mixtures

We followed an approach similar to that recently used to determine the number and stabilities of ternary Cu(GHK)N_Im_^HSA^ complexes formed between human serum albumin (HSA) and glycyl-L-histidyl-L-lysine (GHK) [26]. The Cu(PBT2)N_Im_^Aβ^ spectrum was assumed to be a superposition of the spectra of two ternary complexes. The first complex involved anchoring of Cu(PBT2) on the His6 side chain (N_Im_^H6^), which is also required for Cu(Aβ) formation; the second involved anchoring on a side chain of either His13 or His14 (N_Im_^H13/14^), which is not required for Cu(Aβ) or Cu_2_(Aβ) formation. Thus, Cu(Aβ) and Cu_2_(Aβ) can simultaneously accommodate an Cu(PBT2)N_Im_^H13/14^ complex but not a Cu(PBT2)N_Im_^H6^ complex. Under these assumptions, Cu/PBT2/Aβ_1–40_ *n*:1:1 can be modeled as Cu/L/A/B *n*:1:1:1, where ligand L is PBT2, ligand A acts like Aβ, and ligand B is treated as an isolated N_Im_^H13/14^ donor. The relevant formation constants are:

(2) β′1100=KCuLCuc        for Cu(PBT2)β′1200=KCuLCuc×KCuL2CuLc  for CuPBT22β′1010=KCuACuc       for Cu(Aβ)β′1020=KCuACuc×KCu2ACuAc  for Cu2(Aβ)β′1110=KCuLCuc×KCuLACuLc  for Cu(PBT2)NImH6β′1101=KCuLCuc×KCuLBCuLc  for Cu(PBT2)NImH13/14

where the cumulative conditional (pH-dependent) constants ($\beta^{\prime }$) are defined by

(3) $${\beta^{\prime }}_{pqrs}=\frac{\left[{\mathrm{Cu}}_{p}L_{q}A_{r}B_{s}\right]}{{\left[\mathrm{Cu}\right]}^{p}{\left[L\right]}^{q}{\left[A\right]}^{r}{\left[B\right]}^{s}}\;\mathrm{for}\;p\mathrm{Cu}+qL+rA+sB\;\overset{\beta_{pqrs}}{⇌}\;{\mathrm{Cu}}_{p}L_{q}A_{r}B_{s}$$

and the stepwise conditional formation constants (Kc) are defined by

(4) KCuXCuc=[CuX]Cu[X]  for   Cu+X ⇌KCuXCucCuX (X = L, A)KCuLXCuLc=[CuLX]CuL[X]  for   CuL+X ⇌KCuLXCuLcCuLX (X = A, B)KCu2ACuAc=[Cu2A]CuA[Cu] for      CuA+Cu ⇌KCu2ACuAcCu2A.

To determine the ternary formation constants KCuLACuLc and KCuLBCuLc, we made use of the previously published values for KCuLCuc,KCuL2CuLc,KCuACuc, and KCu2ACuAc at pH 7.4 (Table 2 and Table S2) Initial guesses for KCuLACuLc and KCuLBCuLc were then made, and their values were systematically varied as follows:

(1) For each value of KCuLACuLc and KCuLBCuLc, the theoretical distributions of CuL, CuL_2_, CuA, Cu_2_A, CuLA, and CuLB were calculated for the condition Cu/PBT2/Aβ_1–40_ 1:1:1 ≡ Cu/L/A/B 1:1:1:1 under which spectral features attributable to the ternary species were maximal (Figure 2c).
(2) The theoretical speciation in step 1 provided weighting factors that were used to algebraically subtract the normalized spectra of CuL, CuL_2_, and CuA (Figure 2b) from the experimental spectrum of Cu/PBT2/Aβ_1–40_ 1:1:1 ≡ Cu/L/A/B 1:1:1:1, thus yielding a weighted summation of indistinguishable CuLA and CuLB spectra.
(3) Linear combinations of the normalized CuL, CuL_2_, CuA, Cu_2_A, CuLA, CuLB basis spectra were used to reconstruct the experimental EPR spectra at all intermediate stoichiometries Cu/PBT2/Aβ_1–40_ *n*:1:1 ≡ Cu/L/A/B *n*:1:1:1 (0.33 ≤ *n* ≤ 2.67), and the weightings were iteratively varied using a generalized reduced gradient nonlinear solver (Frontline Systems Inc., Incline Village, NV, USA) to minimize the root-mean-squared deviation between the reconstructions and the experimental spectra.
(4) The deviation between the fitted and experimental values of [CuL], [CuL_2_], [CuA], [Cu_2_A], and [CuLN_Im_^Aβ^] = [CuLA] + [CuLB] for all values of *n* was calculated.
(5) New values of KCuLACuLc and KCuLBCuLc were chosen and steps 1–4 were repeated until the root-mean-squared deviation was minimized.

The above method assumed that the frozen-solution EPR spectra of Cu(PBT2)N_Im_^H6^ and Cu(PBT2)N_Im_^H13/14^ are indistinguishable, which is justified by the identification of a common set of spin Hamiltonian parameters for numerous Cu(PBT2)N_Im_ complexes with different N_Im_ donor ligands (Table 1 and Table S1). This assumption was also shown to be true for ternary Cu^2+^ complexes of different N_Im_ donors with other terdentate ligands such as the non-chlorinated homologue of PBT2 (Table S1), the GHK tripeptide [16,28], and α-synuclein [29].

## Figures and Tables

**Table 1:** Principal electron Zeeman (*g*_z_) and nuclear hyperfine (*A*_z_) parameters of binary and ternary Cu^2+^ complexes of PBT2 and its non-chlorinated homologue with Aβ_1–40_ and other imidazole-bearing (N_Im_) co-ligands. A detailed comparison with previously obtained parameters is shown in Table S1.

Complex			*g_z_*	*A_z_* (^63^Cu) *^a^*	Reference
L = PBT2					
	CuL		2.259 ± 0.002	151 ± 1	This work *^b^*
	CuL_2_		2.283 ± 0.002	148 ± 3	This work *^b^*
	CuLN_Im_^X^				
		X = Aβ_1–40_	2.249 ± 0.002	147 ± 2	This work *^b^*
		X = imidazole	2.248 ± 0.001	143 ± 1	This work *^b^*
		X = histamine	2.248 ± 0.001	143 ± 1	This work *^b^*
		X = Aβ_1–42_	2.242 ± 0.002	142 ± 3	10 *^c^*
L = non-chlorinated PBT2 homologue					
	CuL		2.255 ± 0.001	153 ± 1	6
	CuL_2_		2.267 ± 0.001	149 ± 1	6
	CuLN_Im_^X^				
		X = imidazole	2.245 ± 0.001	144 ± 1	This work *^b^*, 6, 11
		X = histamine	2.245 ± 0.001	145 ± 1	This work *^b^*, 6, 11
Aβ					
	Cu(Aβ_1–40_)		2.268 ± 0.002	174 ± 2	This work *^d^*
	Cu_2_(Aβ_1–40_)				
		first site	2.268 ± 0.002	174 ± 2	This work *^e^*
		second site	2.309 ± 0.005	168 ± 5	This work *^e^*

*^a^* Hyperfine parameters are expressed in units of 10^−4^ cm^−1^ (1 × 10^−4^ cm^−1^ = 2.9979 MHz). *^b^* To aid comparison with other studies, hyperfine couplings have been converted from those obtained using ^65^Cu to those expected for ^63^Cu using the scaling factor |*g*_n_(^65^Cu)/*g*_n_(^63^Cu)| = 1.07. *^c^* Species was not assigned to a ternary complex and spectral isolation was approximate. *^d^* Parameters are those for the dominant species at pH 7.4. *^e^* Coordination of the first-bound ion at the “first site” is assumed to be unperturbed by binding of a second ion at the “second site”.

**Table 2:** Stepwise conditional formation constants (pH 7.4) used to simulate the species distributions of Cu/L/N_Im_^X^ mixtures for L = PBT2 and X = Aβ_1–40_ (Figure 2c), imidazole (Figure S6c), and histamine (Figure S9c). A detailed comparison of Cu^2+^ formation constants, including those for the non-chlorinated homologue of PBT2 and various N_Im_ donors, is provided in Table S2.

Complex	Formation Constant ^*a*^	log[Kc/(1 M^−1^)] at pH 7.4	Reference
CuL	KCuLCuc	13.61 ± 0.05	8
CuL_2_	KCuL2CuLc	5.95 ± 0.07	8
CuLN_Im_^Aβ^ (His6)	KCuLNImH6CuLc	6.4 ± 0.1	This work
CuLN_Im_^Aβ^ (His13/14)	KCuLNImH13/14CuLc	4.4 ± 0.1	This work
CuLN_Im_^imidazole^	KCuLNImimidazoleCuLc	4.22 ± 0.09	This work
CuLN_Im_^histamine^	KCuLNImhistamineCuLc	4.00 ± 0.05	This work
Cu(Aβ_1–40_)	KCu(Aβ)Cuc	10.0 ± 0.1	This work
Cu_2_(Aβ_1–40_)	KCu2(Aβ)Cu(Aβ)c	8.0 ± 0.1	This work

*^a^* Formation constants are defined in Equations (2)–(4) (Section 4.3).

## Data Availability

The data presented in this study are available on request from the corresponding author.

## Supplementary Materials

The following supporting information can be downloaded at: https://www.mdpi.com/article/10.3390/ijms24119267/s1. References [30,31,32,33] are cited in the supplementary materials.
