# Supplementary material for: Chelator PBT2 Forms a Ternary Cu2+ Complex with β-Amyloid That Has High Stability but Low Specificity
Source: Int J Mol Sci. 2023 May 25;24(11):9267. doi: 10.3390/ijms24119267 (PMC10252752; doi:10.3390/ijms24119267)
Supplement: Supplementary file 1 [file ijms-24-09267-s001.zip › ijms-2403348-supplementary.pdf]

Supporting Information for:

Chelator PBT2 Forms a Ternary Cu2+ Complex with β-Amyloid That Has High Stability but Low Specificity

Simon C. Drew [1,2]

1 Brain–Immune Communication Lab, Institut Pasteur, Université Paris Cité, F-75015 Paris, France; sdrew@pasteur.fr or sdrew@unimelb.edu.au

2 Department of Medicine (Royal Melbourne Hospital), The University of Melbourne, Melbourne, VIC 3010, Australia

Table S1. Principal electron Zeeman (g_z) and nuclear hyperfine (A_z) parameters of binary and ternary Cu^{2+} complexes of PBT2 (5,7-dichloro-2-[(dimethylamino)methyl]-8-hydroxyquinoline) and its non-chlorinated homologue (2-[(dimethylamino)methyl]-8-hydroxyquinoline) with imidazole-bearing co-ligands (N_{Im}) from free imidazole, histamine, L-His, and His side chains of proteins and peptides.

Complex	g_z	A_z (^{63}Cu) [a]	Conditions	Reference
**L = PBT2**				
CuL	2.259 ± 0.002	151 ± 1	PBS, pH 7.4	This work,6[b,c]
	2.261 ± 0.003	150 ± 3	"aqueous", pH ≈ 6.5	8
	2.269	151	DMSO	7[d]
CuL_2	2.283 ± 0.002	148 ± 3	PBS, pH 7.4	This work[b]
	2.208 ± 0.003	141 ± 1	"aqueous", pH ≈ 6.5	8
	2.272 ± 0.002	147 ± 9	MOPS/DTAB, pH 7.4	10[f]
	2.275 ± 0.002	147 ± 9	DMSO	7
$CuLN_{Im}^X$				
X = $A\beta_{1\text{–}40}$	2.249 ± 0.002	147 ± 2	PBS, pH 7.4	This work[b]
X = imidazole	2.248 ± 0.001	143 ± 1	PBS, pH 6.9	This work,6[b]
X = histamine	2.248 ± 0.001	143 ± 1	PBS, pH 6.9	This work[b,c]
X = L-His	2.250 ± 0.003	149 ± 1	"aqueous", pH ≈ 6	8[e]
X = $A\beta_{1\text{–}42}$	2.242 ± 0.002	142 ± 3	MOPS/DTAB, pH 7.4	10[f,g]
**L = non-chlorinated PBT2 homologue**				
CuL	2.255 ± 0.001	153 ± 1	PBS, 0.22% v/v DMSO, pH 6.9	6[b]
	2.255 ± 0.001	155 ± 1	PBS, 0.22% v/v DMSO, pH 6.9	This work[b]
CuL_2	2.267 ± 0.001	149 ± 1	PBS, 0.22% v/v DMSO, pH 6.9	6[b]
	2.267 ± 0.001	148 ± 1	PBS, 0.22% v/v DMSO, pH 6.9	This work[b]
$CuLN_{Im}^X$				
X = any	2.245 ± 0.001	145 ± 1	PBS, 0.22% v/v DMSO, pH 6.9	6,11[b]
X = imidazole	2.245 ± 0.001	144 ± 1	PBS, 0.22% v/v DMSO, pH 6.9	This work[b]
X = histamine	2.245 ± 0.001	145 ± 1	PBS, 0.22% v/v DMSO, pH 6.9	This work[b]
**Aβ**				
$CuA\beta_{1\text{–}40/42}$	2.268 ± 0.005	174 ± 3	PBS, pH 7.4	This work[b,h]
$CuA\beta_{1\text{–}16}$	2.272 ± 0.005	171 ± 3	PBS, pH 6.3–8.0	14[b,h]
$Cu_2(A\beta_{1\text{–}40/42})$				
first site	2.268 ± 0.005	174 ± 3	PBS, pH 7.4	This work[b,h,i]
second site	2.309 ± 0.005	168 ± 5	PBS, pH 7.4	This work[b,i]
$Cu_2(A\beta_{1\text{–}28})$				
first site	2.27	170	10 mM PB,160 mM NaCl, pH7.4	30[d,i,j]
second site	2.28	175	10 mM PB,160 mM NaCl, pH7.4	30[d,i,j]
**imidazole**				
$Cu(imidazole)_4$	2.254 ± 0.001	190 ± 1	PBS, pH 6.9	This work
**histamine**				
$Cu(histamine)_2$	2.237 ± 0.001	191 ± 1	PBS, pH 6.9	This work

[a] All hyperfine parameters are expressed in units of 10^{-4}cm^{-1} (1 × 10^{-4}cm^{-1} = 2.9979 MHz). [b] To aid comparison with other studies, hyperfine couplings have been converted from those obtained using ^{65}Cu to those expected for ^{63}Cu using the scaling factor $|g_n(^{65}Cu) / g_n(^{63}Cu)| = 1.07$. [c] See Figure S33 of ref. [6]. [d] Natural abundance ^{63,65}Cu was used, therefore value of $A_{\parallel}(^{63}Cu)$ is approximate. [e] Ternary complex correctly identified but denticity incorrectly assigned. [f] MOPS/DTAB = 100 mM 3-(N-morpholino)propanesulfonic acid, 400 mM dodecyl trimethylammonium bromide. [g] Species was not assigned to a ternary complex. [h] Parameters are those of the dominant coordination mode (component Ia/b [14]). [i] Coordination of the first-bound ion at the "first site" is assumed to be unperturbed by binding of a second ion at the "second site". See Figure S2 for details. [j] Erroneous conversion of hyperfine value in the original reference has been corrected.

Table S2. Conditional stepwise formation constants for relevant Cu^{2+} complexes at pH 7.4. Absolute stability constants from potentiometric data have been converted to conditional constants at pH 7.4 using protonation constants in the corresponding reference and correction factors in Table S3.

Complex	Stepwise formation constant	log[^cK /(1 M^{-1})] at pH 7.4	Method	Reference
L = PBT2				
CuL	$^cK^{Cu}_{CuL}$	13.61 ± 0.05	UV-vis, 298 K	8
CuL_2	$^cK^{CuL}_{CuL_2}$	5.95 ± 0.07	UV-vis, 298 K	8
$CuLN_{Im}{}^X$				
X = $A\beta_{1-40}$ (His6)	$^cK^{CuL}_{CuLN^{H6}_{Im}}$	6.4 ± 0.1	EPR, 77 K	This work
X = $A\beta_{1-40}$ (His13/His14)	$^cK^{CuL}_{CuLN^{H13/14}_{Im}}$	4.4 ± 0.1	EPR, 77 K	This work
X = imidazole	$^cK^{CuL}_{CuLN^{imidazole}_{Im}}$	4.22 ± 0.09	EPR, 77 K	This work[a]
X = histamine	$^cK^{CuL}_{CuLN^{histamine}_{Im}}$	4.00 ± 0.05	EPR, 77 K	This work[a]
L = non-chlorinated PBT2 homologue				
CuL	$^cK^{Cu}_{CuL}$	10.45 ± 0.03	Potentiometry	6,11
CuL_2	$^cK^{CuL}_{CuL_2}$	4.56 ± 0.06	Potentiometry	6,11
$CuLN_{Im}{}^X$				
X = imidazole	$^cK^{CuL}_{CuLN^{imidazole}_{Im}}$	3.85 ± 0.04	Potentiometry	11
		3.79 ± 0.05	EPR, 77 K	This work[a]
X = histamine	$^cK^{CuL}_{CuLN^{histamine}_{Im}}$	3.74 ± 0.05	EPR, 77 K	This work[a]
Aβ (high affinity)	$^cK^{Cu}_{Cu(A\beta)}$			
$Cu(A\beta_{1-16})$		10.23	Potentiometry	31
		10.0 ± 0.1	Fluorescence	15
$Cu(A\beta_{1-40})$		10.0 ± 0.1	EPR, 77 K	This work
Aβ (low-affinity)	$^cK^{Cu(A\beta)}_{Cu_2(A\beta)}$			
$Cu_2(A\beta_{1-16})$ second site		7.7–8.0	Fluorescence	15
$Cu_2(A\beta_{1-40})$ second site		8.0 ± 0.1	EPR, 77 K	This work
Histamine (HA)				
Cu(HA)	$^cK^{Cu}_{Cu(HA)}$	7.13 ± 0.08	Potentiometry	32
$Cu(HA)_2$	$^cK^{Cu(HA)}_{Cu(HA)_2}$	4.07 ± 0.12	Potentiometry	32
Imidazole (Im)				
Cu(Im)	$^cK^{Cu}_{Cu(Im)}$	4.04 ± 0.07	Potentiometry	32
$Cu(Im)_2$	$^cK^{Cu(Im)}_{Cu(Im)_2}$	3.35 ± 0.07	Potentiometry	32
$Cu(Im)_3$	$^cK^{Cu(Im)_2}_{Cu(Im)_3}$	2.97 ± 0.07	Potentiometry	32
$Cu(Im)_4$	$^cK^{Cu(Im)_3}_{Cu(Im)_4}$	2.09 ± 0.07	Potentiometry	32

[a] Conditional formation constant was converted from the experimental value determined at pH 6.9 to a value at pH 7.4 (Table S3).

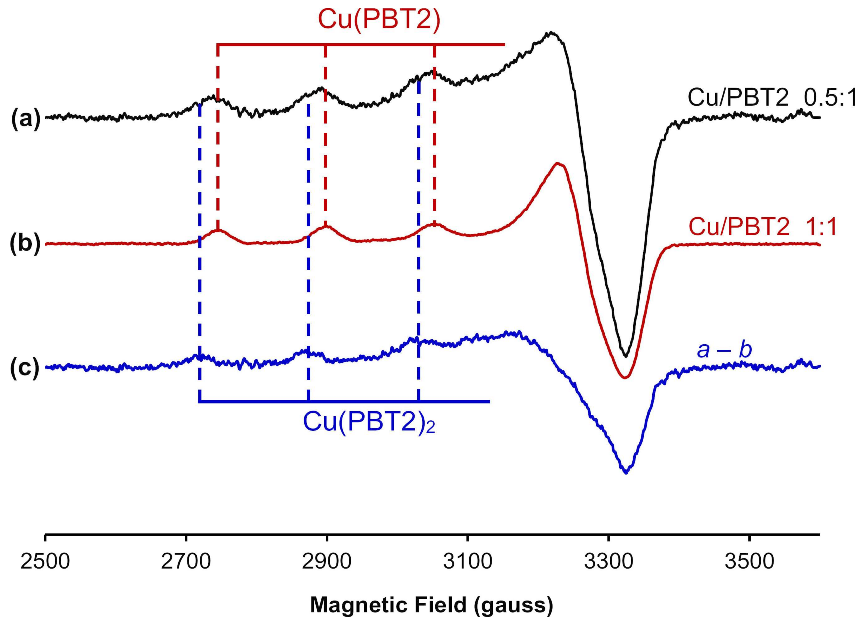

Figure S1. Isolation of the frozen-solution (77 K) X-band (9.425 GHz) EPR spectrum of Cu(PBT2)$_2$. A mixture of Cu/PBT2 <1 yields a spectrum **(a)** with two components, whereas mixtures with Cu/PBT2 ≥1 yield a spectrum **(b)** of pure Cu(PBT2). Following normalization of spectra by double integration, a spectrum **(c)** corresponding to Cu(PBT2)$_2$ was obtained by subtracting **(b)** 45% Cu/PBT2 1:1. Spectra were obtained at pH 7.4 using 10% v/v glycerol and 0.2 mM PBT2.

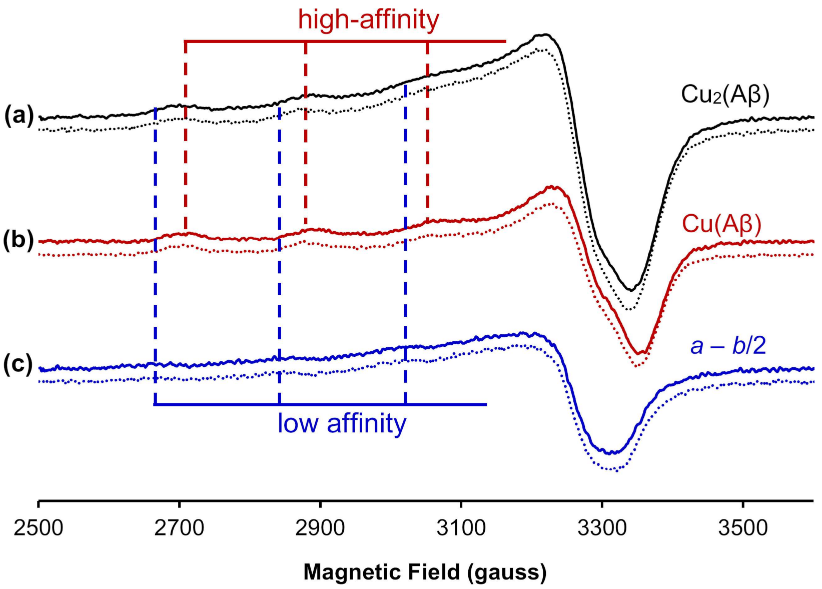

Figure S2. Putative isolation of the frozen-solution (77 K) X-band (9.425 GHz) EPR spectrum of the "low-affinity site" of Aβ_{1-40} (solid lines) and Aβ_{1-42} (dotted lines). After normalisation of the spectra of **(a)** Cu/A$\beta_{1-40/42}$ 2.5:1 and **(b)** Cu/A$\beta_{1-40/42}$ 1:1, 50% spectrum *b* was subtracted from spectrum *a* to isolate **(c)** a putative spectrum of the "second site". Greater than 2 equiv Cu^{2+} were used in panel *a* to promote saturation of the "second site". Any unbound (aqueous) Cu^{2+} will largely precipitate as EPR-silent $[Cu(OH)_2]_n$ at pH 7.4. The isolation assumes that sequential binding of the second Cu^{2+} ion does not change the first coordination sphere or affect the linewidth of the first-bound ion and ignores potential broadening of the spectrum of the first site by dipolar interactions with the second site.

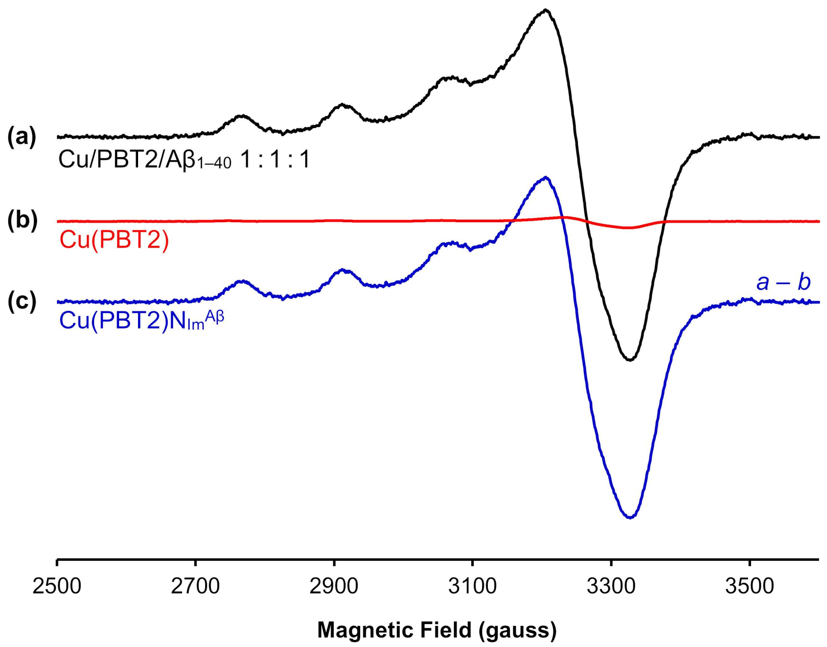

Figure S3. Isolation of the frozen-solution (77 K) X-band (9.425 GHz) EPR spectrum of Cu(PBT2)$N_{Im}^{A\beta}$. A mixture of Cu/PBT2/A$\beta_{1\text{–}40}$ 1:1:1 yields spectrum **(a)**. Using the species distributions (Figure 2c, main text) resulting from the conditional binding constants for ${}^{c}K^{\mathrm{Cu}}_{\mathrm{Cu(A\beta)}}$, ${}^{c}K^{\mathrm{Cu}}_{\mathrm{Cu_2(A\beta)}}$, ${}^{c}K^{\mathrm{Cu(PBT2)}}_{\mathrm{Cu(PBT2)_2}}$, ${}^{c}K^{\mathrm{Cu(PBT2)}}_{\mathrm{Cu(PBT2)N^{H6}_{Im}}}$ and ${}^{c}K^{\mathrm{Cu(PBT2)}}_{\mathrm{Cu(PBT2)N^{H13/14}_{Im}}}$ (Table 2, main text), spectrum **(c)** corresponding to Cu(PBT2)$N_{Im}^{A\beta}$ = Cu(PBT2)N_{Im}^{H6} + Cu(PBT2)$N_{Im}^{H13/14}$ is obtained after subtracting **(b)** $\approx$ 2% Cu(PBT2). At the above stoichiometry, the percentages of all other species are approximately zero (Table 1, Figure 2b; main text).

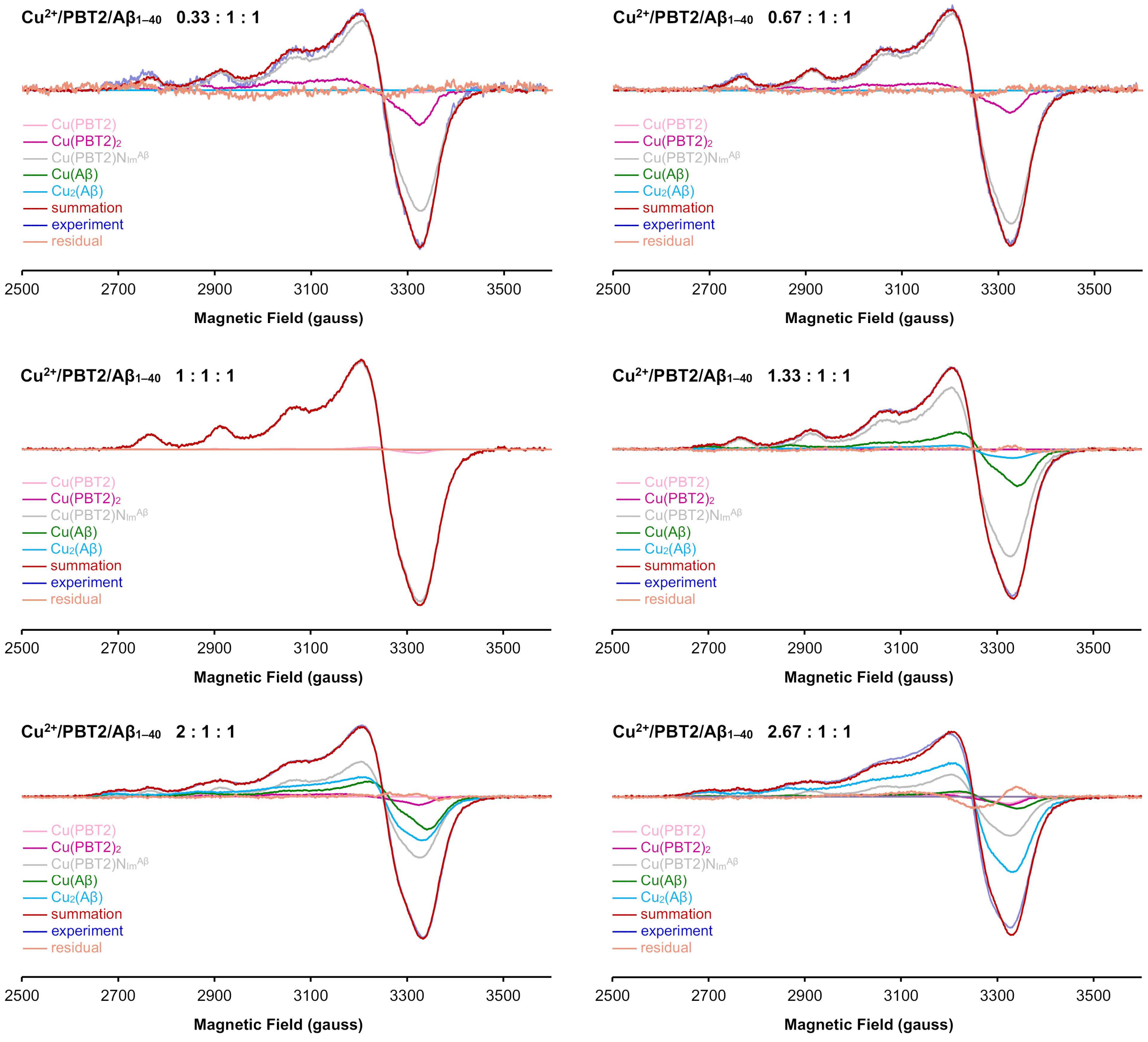

Figure S4. Decomposition of the frozen-solution (77 K) X-band (9.425 GHz) EPR spectra of ^{65}Cu/PBT2/A$\beta_{1\text{–}40}$ *n* : 1 : 1 (*n* = 0.33–2.67). The corresponding percentages of each species are shown in Figure 2 (main text). *Blue spectrum*, experiment; *pink spectrum*, Cu(PBT2); *violet spectrum*, Cu(PBT2)$_2$; *grey spectrum*, Cu(PBT2)N$_{Im}^{A\beta}$; *green spectrum*, Cu(Aβ); *cyan spectrum*, Cu$_2$(Aβ); *red spectrum*, summation of Cu(PBT2), Cu(PBT2)$_2$, Cu(PBT2)N$_{Im}^{A\beta}$, Cu(Aβ), and Cu$_2$(Aβ) spectra; *orange spectrum*, difference between the experimental spectrum (blue) and the summation (red). The largest difference is seen at 2.67 equiv Cu^{2+}, which can be explained by dipolar interactions between the Cu(PBT2)N$_{Im}^{H13/14}$ complex and Cu$_2$(Aβ) upon which it is anchored. These interactions can cause broadening of the experimental spectra that is not accounted for by a linear superposition of Cu$_2$(Aβ) (Figure S2) and Cu(PBT2)N$_{Im}^{A\beta}$ (Figure S3) spectra.

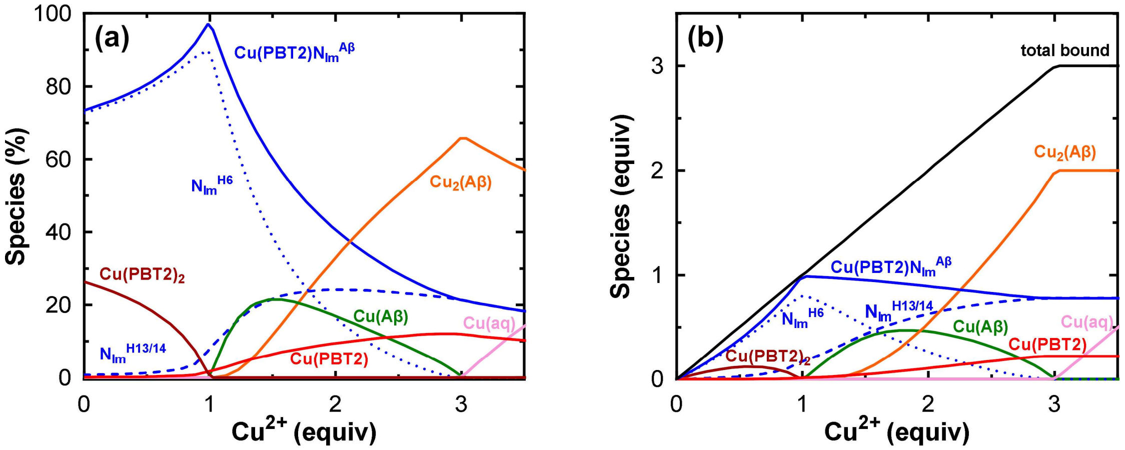

Figure S5. Theoretical species distributions for the Cu/PBT2/Aβ_{1-40} *n*:1:1 system calculated using the relevant formation constants in Table 2 (main text) and presented as **(a)** a percentage of total Cu^{2+} and **(b)** the number of Cu^{2+} equivalents. The solid blue line shows the sum of the Cu(PBT2)N_{Im}^{H6} (dotted line) and Cu(PBT2)$N_{Im}^{H13/14}$ (dashed line) distributions. Cu(aq) denotes aqueous Cu^{2+}. The black line in panel *b* shows the sum of all species other than Cu(aq). Assignment of the low-affinity ternary complex to Cu(PBT2)$N_{Im}^{H13/14}$ assumed that His6 remains Cu^{2+}-bound and His13 or His14 remains unbound at all Cu^{2+}/Aβ ratios.

Analysis of Cu/L/N_{Im}^{X} (X = imidazole, histamine; L = PBT2 and its non-chlorinated homologue)

Previous analyses using the non-chlorinated PBT2 homologue were performed at pH 6.9 [6], and those using PBT2 were done at the same pH for comparison. Stock solutions of L, X, and then $^{65}CuCl_2$ were combined to achieve final molar Cu/L/X ratios of 1:1:*n* (*n* = 0–20) in PBS, the pH was adjusted to 6.9, then the samples were transferred to quartz EPR tubes and snap frozen in liquid nitrogen. To prevent formation of a concentrated solute phase of the low-molecular-weight binary and ternary complexes, 10% v/v glycerol was added prior to pH adjustment and freezing.

The conditional (pH-dependent) formation constants ${}^{c}K_{\mathrm{CuL}}^{\mathrm{Cu}}$, and ${}^{c}K_{\mathrm{CuL_2}}^{\mathrm{Cu}}$ for L = PBT2 were determined at pH 7.4 [8] (Table 2, main text) but can be adjusted to their values at pH 6.9 by using the Schwarzenbach alpha coefficients [34]. For the stepwise equilibria

$$\mathrm{Cu} + \mathrm{A} \overset{K_{\mathrm{CuA}}^{\mathrm{Cu}}}{\rightleftharpoons} \mathrm{CuA}$$
$$\mathrm{CuA} + \mathrm{B} \overset{K_{\mathrm{CuAB}}^{\mathrm{CuA}}}{\rightleftharpoons} \mathrm{CuAB} \qquad \text{(S1)}$$

the conditional formation constants are defined as:

$${}^{c}K_{\mathrm{CuA}}^{\mathrm{Cu}} = K_{\mathrm{CuA}}^{\mathrm{Cu}}/\alpha_{\mathrm{A}}$$
$${}^{c}K_{\mathrm{CuAB}}^{\mathrm{CuA}} = K_{\mathrm{CuAB}}^{\mathrm{CuA}}/\alpha_{\mathrm{B}} \qquad \text{(S2)}$$

where $K_{\mathrm{CuA}}^{\mathrm{Cu}}$ and $K_{\mathrm{CuAB}}^{\mathrm{CuA}}$ are the absolute (pH-independent) stability constants and the alpha coefficients are

$$\alpha_{\mathrm{A}} = 1 + 10^{-(\mathrm{pH}-\mathrm{p}K_{\mathrm{a1}}^{\mathrm{A}})} + 10^{-(2\mathrm{pH}-\mathrm{p}K_{\mathrm{a1}}^{\mathrm{A}}-\mathrm{p}K_{\mathrm{a2}}^{\mathrm{A}})} \ldots + 10^{-(n_{\mathrm{A}}\mathrm{pH}-\mathrm{p}K_{\mathrm{a1}}^{\mathrm{A}}-\mathrm{p}K_{\mathrm{a2}}^{\mathrm{A}}-\mathrm{p}K_{\mathrm{a3}}^{\mathrm{A}}\cdots-\mathrm{p}K_{\mathrm{a}n_{\mathrm{A}}}^{\mathrm{A}})}$$
$$\alpha_{\mathrm{B}} = 1 + 10^{-(\mathrm{pH}-\mathrm{p}K_{\mathrm{a1}}^{\mathrm{B}})} + 10^{-(2\mathrm{pH}-\mathrm{p}K_{\mathrm{a1}}^{\mathrm{B}}-\mathrm{p}K_{\mathrm{a2}}^{\mathrm{B}})} \ldots + 10^{-(n_{\mathrm{B}}\mathrm{pH}-\mathrm{p}K_{\mathrm{a1}}^{\mathrm{B}}-\mathrm{p}K_{\mathrm{a2}}^{\mathrm{B}}-\mathrm{p}K_{\mathrm{a3}}^{\mathrm{B}}\cdots-\mathrm{p}K_{\mathrm{a}n_{\mathrm{B}}}^{\mathrm{B}})} \qquad \text{(S3)}$$

and $\mathrm{p}K_{\mathrm{a}1}^{\mathrm{A}}, \ldots \mathrm{p}K_{\mathrm{a}n_{\mathrm{A}}}^{\mathrm{A}}$ and $\mathrm{p}K_{\mathrm{a}1}^{\mathrm{B}}, \ldots \mathrm{p}K_{\mathrm{a}n_{\mathrm{B}}}^{\mathrm{B}}$ are the stepwise protonation constants (in order of decreasing magnitude) of ligands A and B, respectively.

To convert the absolute stability constants to conditional formation constants, one takes the logarithm of equations S2:

$$\log {}^{c}K_{\mathrm{CuA}}^{\mathrm{Cu}} = \log K_{\mathrm{CuA}}^{\mathrm{Cu}} - \log\alpha_{\mathrm{A}}$$
$$\log {}^{c}K_{\mathrm{CuAB}}^{\mathrm{CuA}} = \log K_{\mathrm{CuAB}}^{\mathrm{Cu}} - \log\alpha_{\mathrm{B}} \qquad \text{(S4)}$$

Using equations 3, the conditional formation constants at one pH value ($\mathrm{pH_1}$) can be related to those at another ($\mathrm{pH_2}$) by calculating the values of α_{A} and α_{B} at each pH:

$$\log {}^{c}K_{\mathrm{CuA}}^{\mathrm{Cu}}(\mathrm{pH_2}) = \log {}^{c}K_{\mathrm{CuA}}^{\mathrm{Cu}}(\mathrm{pH_1}) + \log\alpha_{\mathrm{A}}(\mathrm{pH_1}) - \log\alpha_{\mathrm{A}}(\mathrm{pH_2})$$
$$\log {}^{c}K_{\mathrm{CuAB}}^{\mathrm{CuA}}(\mathrm{pH_2}) = \log {}^{c}K_{\mathrm{CuAB}}^{\mathrm{CuA}}(\mathrm{pH_1}) + \log\alpha_{\mathrm{B}}(\mathrm{pH_1}) - \log\alpha_{\mathrm{B}}(\mathrm{pH_2}) \qquad \text{(S5)}$$

Only deprotonated groups involved in Cu coordination are included in equations S3. For Cu(HA) and $\mathrm{Cu(HA)_2}$, ligand A is the same as ligand B and both coordinate in a bidentate fashion via the deprotonated amine and imidazole groups; thus, $\mathrm{p}K_{\mathrm{a}}$ values of both groups are included in the calculation of $\alpha_{\mathrm{A}} = \alpha_{\mathrm{B}}$. For CuL, the coordination is terdentate and thus $\mathrm{p}K_{\mathrm{a}}$ values of both the exocyclic amine and the phenol group are included in the calculation α. Similarly, $\mathrm{Cu(PBT2)N_{Im}}^{\mathrm{histamine}}$ involves terdentate PBT2 (A) and monodentate $\mathrm{N_{Im}}$ coordination of histamine (B); thus, only the $\mathrm{p}K_{\mathrm{a}}$ value of $\mathrm{N_{Im}}$ is included in the calculation of α_{B}.

Table S3. Schwarzenbach alpha coefficients determined from the protonation constants (K_a) using equations S3. The coefficients were used to convert absolute stability constants to conditional formation constants (^cK) at pH 6.9 or 7.4 using equations S4. They were also used to adjust conditional formation constants from pH 6.9 to 7.4 using equations S5.

Species	Group protonated	Stepwise formation constant		Value	
**L = PBT2**					
			**p*K*a** [8]	**log α (terdentate)**	
HL	exocyclic amine	K^{L}_{HL} ($1/K_{a1}$)	9.49 ± 0.01	**pH 7.4**	**pH 6.9**
H_2L	phenolate	$K^{HL}_{H_2L}$($1/K_{a2}$)	6.48 ± 0.02	2.142	2.731
**L non-chlorinated PBT2 homologue**					
			**p*K*a** [6,11]	**log α (terdentate)**	
HL	exocyclic amine	K^{L}_{HL} ($1/K_{a1}$)	10.13 ± 0.01	**pH 7.4**	**pH 6.9**
H_2L	phenolate	$K^{HL}_{H_2L}$($1/K_{a2}$)	8.39 ± 0.01	3.762	4.734
**L = imidazole**			**p*K*a** [32]	**log α** [a]	
HL	amine	K^{L}_{HL} ($1/K_{a1}$)	7.04 ± 0.02	**pH 7.4**	**pH 6.9**
				0.157	0.377
**L = histamine**			**p*K*a** [32]	**log α (bidentate)**	
HL	amine	K^{L}_{HL} ($1/K_{a1}$)	9.81 ± 0.02	**pH 7.4**	**pH 6.9**
H_2L	imine (N_{Im})	$K^{HL}_{H_2L}$($1/K_{a2}$)	6.07 ± 0.02	2.431	2.970
				**log α (monodentate)** [a]	
				**pH 7.4**	**pH 6.9**
				0.020	0.060

[a] Coordination of imine nitrogen only.

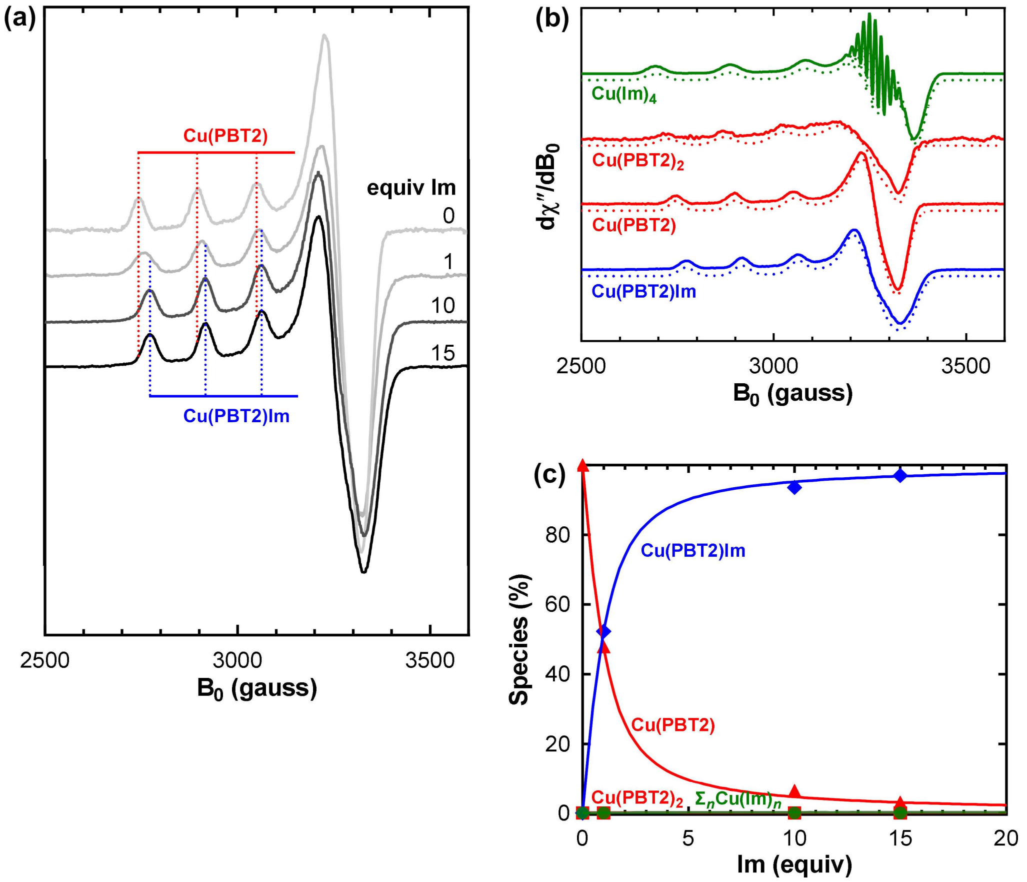

Figure S6. Determination of the stability of the ternary Cu(PBT2)N$_{Im}$^{X} complex formed by X = imidazole (Im). (a) CW-EPR spectra of ^{65}Cu/PBT2/Im 1:1:*n* (*n* = 0–15) at 77 K in PBS pH 6.9 (0.225 mM PBT2). **(b)** Normalized basis set used for the decomposition of the spectra in panel *a*. The spectrum of Cu(Im)$_4$ was obtained using a Cu/histamine ratio of 1:50. The spectra of Cu(PBT2) and Cu(PBT2)$_2$ were obtained as described in Figure S1 and Figure S3. The spectrum of Cu(PBT2)Im was obtained as described in Figure S7. Dotted lines show spectra simulated using the parameters in Table S1. Spectra are drawn on a different scale from those in panel *a*. **(c)** Experimental species distributions (points) resulting from spectral decomposition (Figure S7) and theoretical distributions (lines) calculated using the relevant formation constants in Table S2. The percentages of Cu(PBT2)$_2$ and Cu(Im)$_i$ (i = 1, 2) are zero for all values of *n*. Experimental conditions: temperature, 77 K; microwave power, 10 mW; microwave frequency, 9.425 GHz; modulation amplitude, 8 G; sweep time, 180 s; time constant, 328 ms; averages, 4.

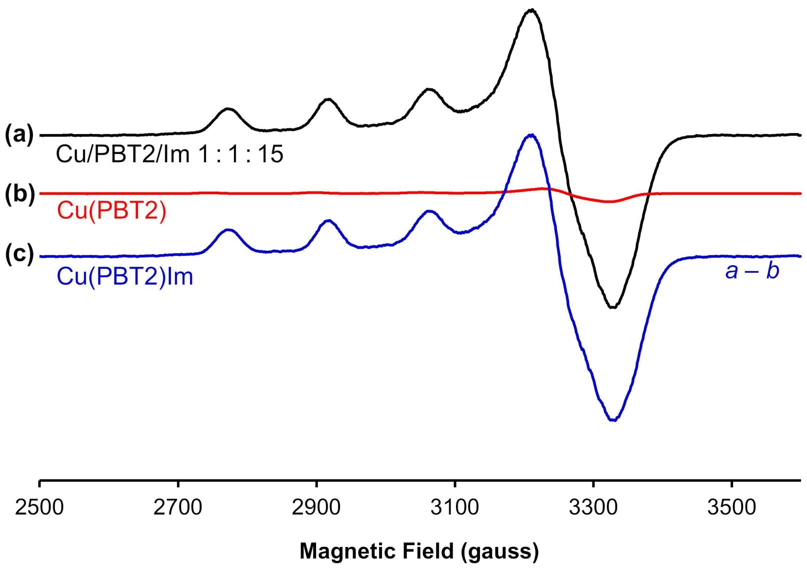

Figure S7. Isolation of the frozen-solution (77 K) X-band (9.425 GHz) EPR spectrum of Cu(PBT2)Im. A mixture of Cu/PBT2/Im 1:1:15 yields spectrum **(a)**. Using the species distributions (Figure S6c) resulting from the conditional binding constants for ${}^{c}K^{\mathrm{Cu}}_{\mathrm{Cu(PBT2)}}$, ${}^{c}K^{\mathrm{Cu(PBT2)}}_{\mathrm{Cu(PBT2)_2}}$, ${}^{c}K^{\mathrm{Cu(PBT2)}}_{\mathrm{Cu(PBT2)Im}}$, and the set of ${}^{c}K^{\mathrm{Cu(Im)}_i}_{\mathrm{Cu(Im)}_{i+1}}$ (i = 0–3) constants (Table S2), subtraction of (b) ≈3% Cu(PBT2) yields a unique spectrum corresponding to (c) Cu(PBT2)Im. At the above stoichiometry, the percentages of all other species are approximately zero; therefore, the spectrum is almost entirely comprised of Cu(PBT2)Im and easily distinguished from that of all other species (Table S1, Figure S6b).

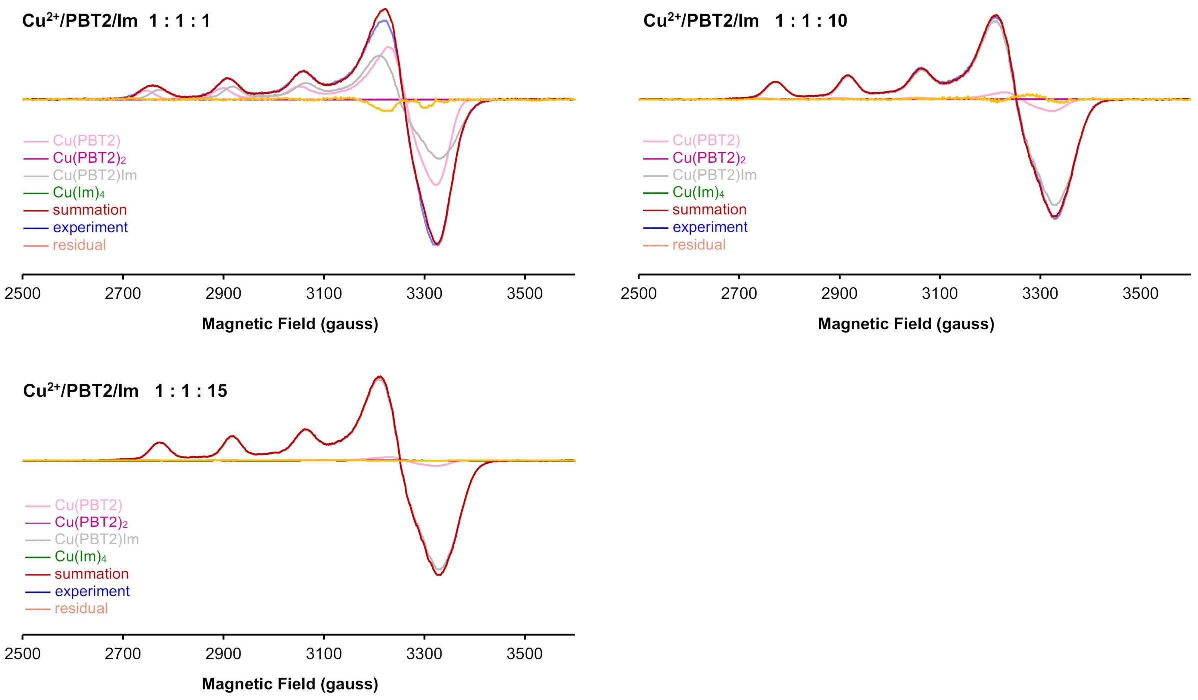

Figure S8. Decomposition of the frozen-solution (77 K) X-band (9.425 GHz) EPR spectra of Cu/PBT2/Im 1 : 1 : *n* (*n* = 1–15). The corresponding percentages of each species are shown in Figure S6c. *Blue spectrum*, experiment; *pink spectrum*, Cu(PBT2); *violet spectrum*, $Cu(PBT2)_2$; *grey spectrum*, Cu(PBT2)Im; *green spectrum*, $Cu(Im)_4$; *red spectrum*, summation of Cu(PBT2), $Cu(PBT2)_2$, Cu(PBT2)Im, and $Cu(Im)_4$ spectra; *orange spectrum*, difference between the experimental spectrum (blue) and the summation (red).

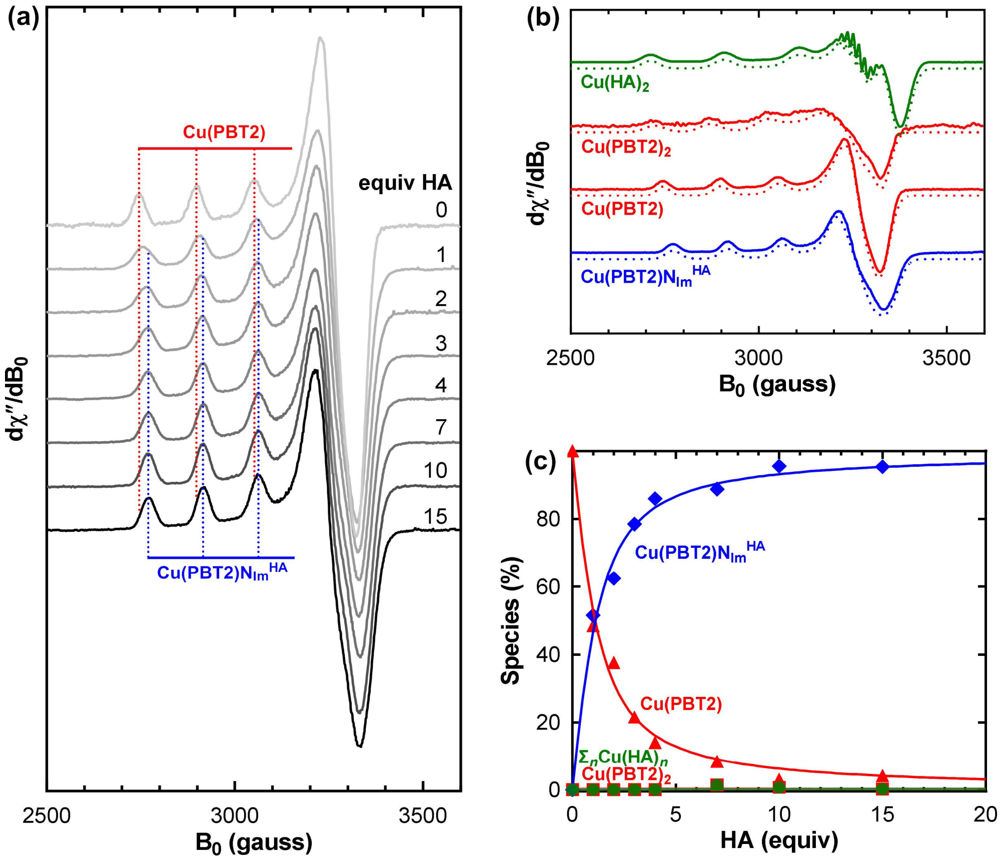

Figure S9. Determination of the stability of the ternary Cu(PBT2)N$_{\mathrm{Im}}^{\mathrm{X}}$ complex formed by X = histamine (HA). (a) CW-EPR spectra of ^{65}Cu/PBT2/HA 1:1:*n* (*n* = 0–15) at 77 K in PBS pH 6.9 (0.225 mM PBT2). **(b)** Normalized basis set used for the decomposition of the spectra in panel *a*. The spectrum of Cu(histamine)$_2$ was obtained using a Cu/histamine ratio of 1:50. The spectra of Cu(PBT2) and Cu(PBT2)$_2$ were obtained as described in Figure S1 and Figure S3. The spectrum of Cu(PBT2)N$_{\mathrm{Im}}^{\mathrm{HA}}$ was obtained as described in Figure S10. Dotted lines show spectra simulated using the parameters in Table S1. Spectra are drawn on a different scale from those in panel *a*. (c) Experimental species distributions (points) resulting from spectral decomposition (Figure S11) and theoretical distributions (lines) calculated using the relevant formation constants in Table S2. The percentages of Cu(PBT2)$_2$ and Cu(HA)$_i$ (i = 1, 2) are zero for all values of *n*. Experimental conditions: temperature, 77 K; microwave power, 10 mW; microwave frequency, 9.425 GHz; modulation amplitude, 8 G; sweep time, 180 s; time constant, 328 ms; averages, 4.

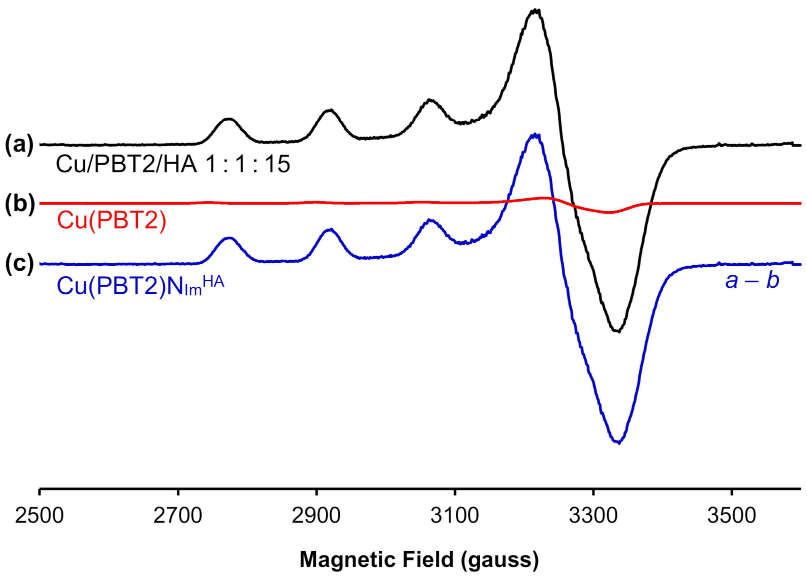

Figure S10. Isolation of the frozen-solution (77 K) X-band (9.425 GHz) EPR spectrum of Cu(PBT2)N$_{\mathrm{Im}}$^{HA}. A mixture of Cu/PBT2/HA 1:1:15 yields spectrum **(a)**. Using the species distributions (Figure S9c) resulting from the conditional binding constants for ${}^{c}K^{\mathrm{Cu}}_{\mathrm{Cu(PBT2)}}$, ${}^{c}K^{\mathrm{Cu(PBT2)}}_{\mathrm{Cu(PBT2)_2}}$, ${}^{c}K^{\mathrm{Cu(PBT2)}}_{\mathrm{Cu(PBT2)N^{HA}_{Im}}}$, ${}^{c}K^{\mathrm{Cu}}_{\mathrm{Cu(HA)}}$, and ${}^{c}K^{\mathrm{Cu(HA)}}_{\mathrm{Cu(HA)_2}}$ (Table S2), subtraction of **(b)** ≈3% Cu(PBT2) yields a unique spectrum corresponding to **(c)** Cu(PBT2)N$_{\mathrm{Im}}$^{HA}. At the above stoichiometry, the percentages of all other species are approximately zero; therefore, the spectrum is almost entirely comprised of Cu(PBT2)N$_{\mathrm{Im}}$^{HA} and easily distinguished from that of all other species (Table S1, Figure S9b).

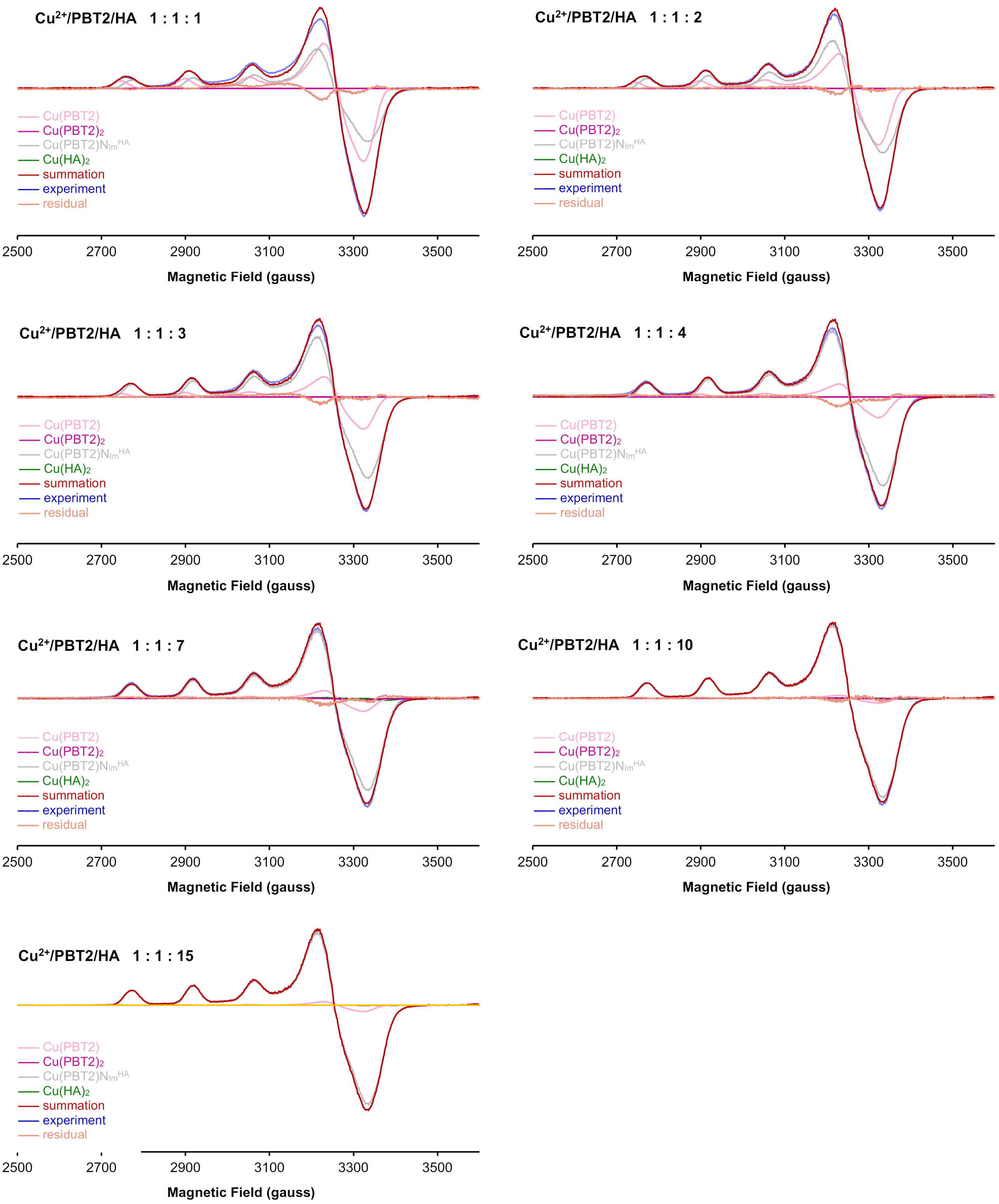

Figure S11. Decomposition of the frozen-solution (77 K) X-band (9.425 GHz) EPR spectra of Cu/PBT2/HA 1 : 1 : *n* (*n* = 1–15). The corresponding percentages of each species are shown in Figure S9c. *Blue spectrum*, experiment; *pink spectrum*, Cu(PBT2); *violet spectrum*, $Cu(PBT2)_2$; *grey spectrum*, $Cu(PBT2)N_{Im}^{HA}$; *green spectrum*, $Cu(HA)_2$; *red spectrum*, summation of Cu(PBT2), $Cu(PBT2)_2$, $Cu(PBT2)N_{Im}^{HA}$, and $Cu(HA)_2$ spectra; *orange spectrum*, difference between the experimental spectrum (blue) and the summation (red).

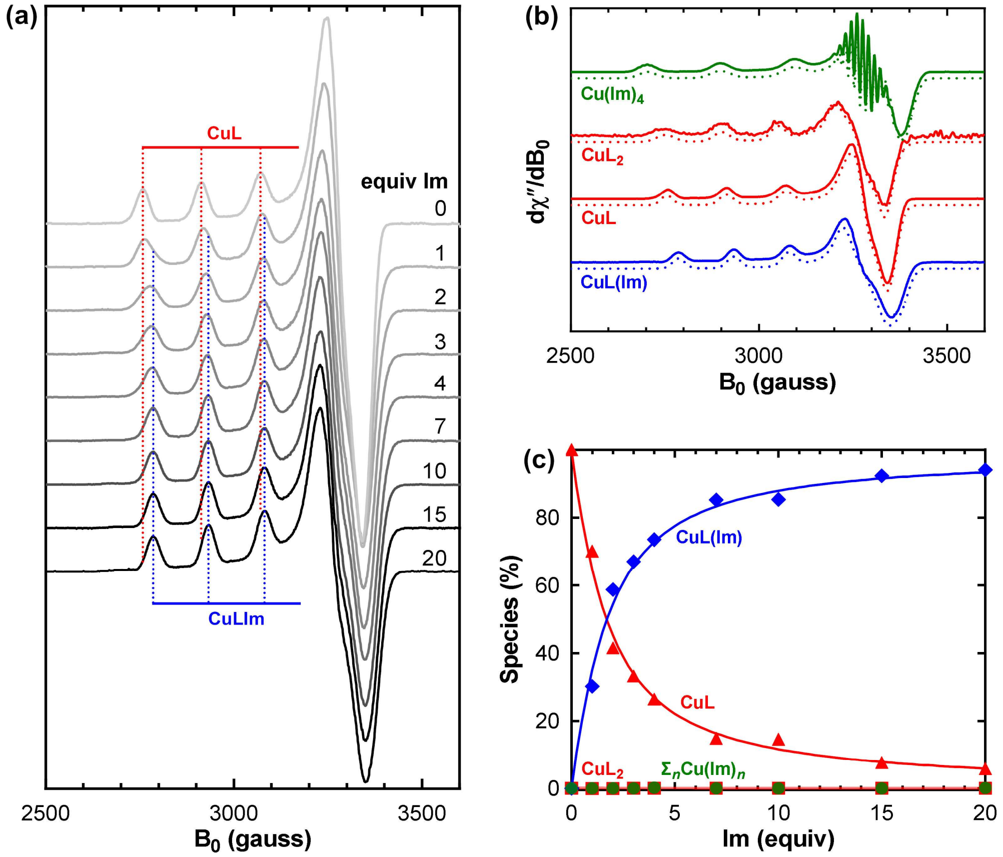

Figure S12. Determination of the stability of the ternary $CuLN_{Im}^{X}$ complex formed by X = imidazole (Im) and the non-chlorinated PBT2 homologue (L). (a) CW-EPR spectra of ^{65}Cu/L/Im 1:1:*n* (*n* = 0–15) at 77 K in PBS pH 6.9 (0.225 mM L), originally reported by Kenche *et al.* [6]. **(b)** Normalized basis set used for the decomposition of the spectra in panel *a*. The spectrum of $Cu(Im)_4$ was obtained using a Cu/Im ratio of 1:50, the spectra of CuL and CuL_2 were obtained as described by Kenche *et al.* [6], and the spectrum of CuL(Im) was obtained as shown in Figure S13. Dotted lines show spectra simulated using the EPR parameters in Table S1. Spectra are drawn on a different scale from those in panel *a*. **(c)** Experimental species distributions (points) resulting from spectral decomposition (Figure S14) and theoretical distributions (lines) calculated using the using the relevant formation constants in Table S2. The percentages of CuL_2 and $Cu(Im)_i$ (i = 1–4) are zero for all values of *n*. Experimental conditions: temperature, 77 K; microwave power, 10 mW; microwave frequency, 9.450 GHz; modulation amplitude, 8 G; sweep time, 180 s; time constant, 328 ms; averages, 4.

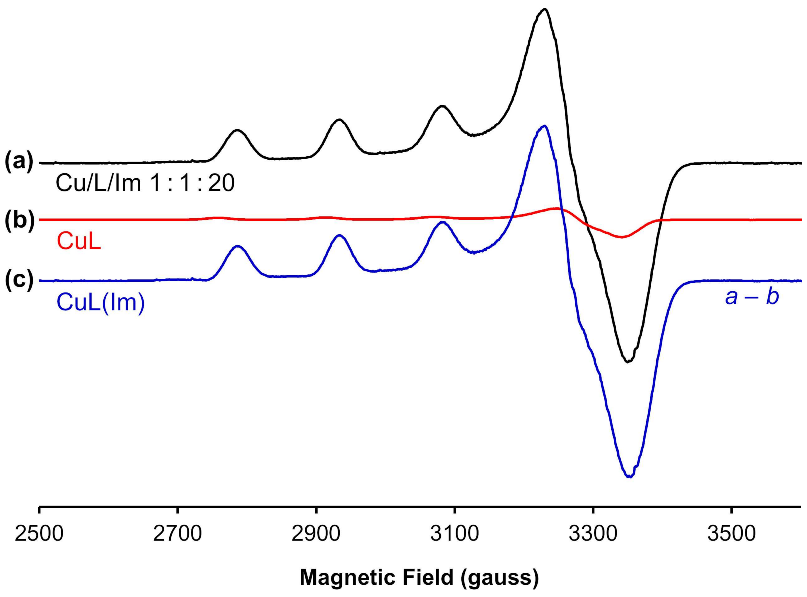

Figure S13. Isolation of the frozen-solution (77 K) X-band (9.450 GHz) EPR spectrum of CuL(Im) for the non-chlorinated PBT2 homologue (L). A mixture of Cu/L/Im 1:1:20 yields spectrum **(a)**. Using the species distributions (Figure S12c) resulting from the conditional binding constants for ${}^{c}K^{\mathrm{Cu}}_{\mathrm{CuL}}$, ${}^{c}K^{\mathrm{CuL}}_{\mathrm{CuL_2}}$, ${}^{c}K^{\mathrm{CuL}}_{\mathrm{CuLIm}}$, and the set of ${}^{c}K^{\mathrm{Cu(Im)}_i}_{\mathrm{Cu(Im)}_{i+1}}$ (i = 0–3) constants (Table S2), subtraction of **(b)** ≈6% CuL yields a unique spectrum corresponding to **(c)** CuL(Im). At the above stoichiometry, the percentages of all other species are approximately zero; therefore, the spectrum is almost entirely comprised of CuL(Im) and easily distinguished from that of all other species (Table S1, Figure S12b).

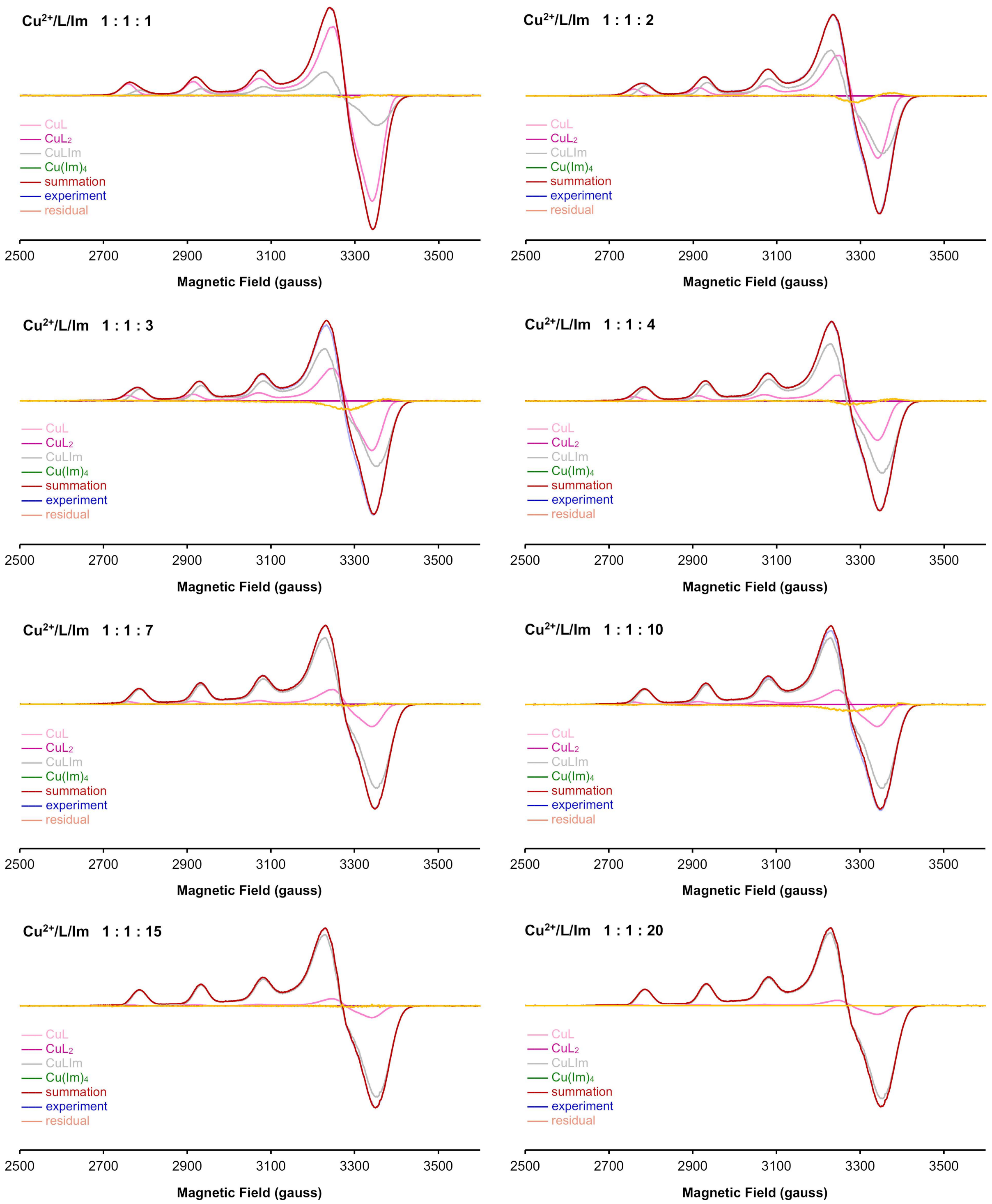

Figure S14. Decomposition of the frozen-solution (77 K) X-band (9.450 GHz) EPR spectra of Cu/L/Im 1 : 1 : *n* (*n* = 1–20) for the non-chlorinated PBT2 homologue (L). The corresponding percentages of each species are shown in Figure S12c. *Blue spectrum*, experiment; *pink spectrum*, CuL; *violet spectrum*, CuL_2; *grey spectrum*, CuLIm; *green spectrum*, $Cu(Im)_4$; *red spectrum*, summation of CuL, CuL_2, CuLIm, and $Cu(Im)_4$ spectra; *orange spectrum*, difference between the experimental spectrum (blue) and the summation (red).

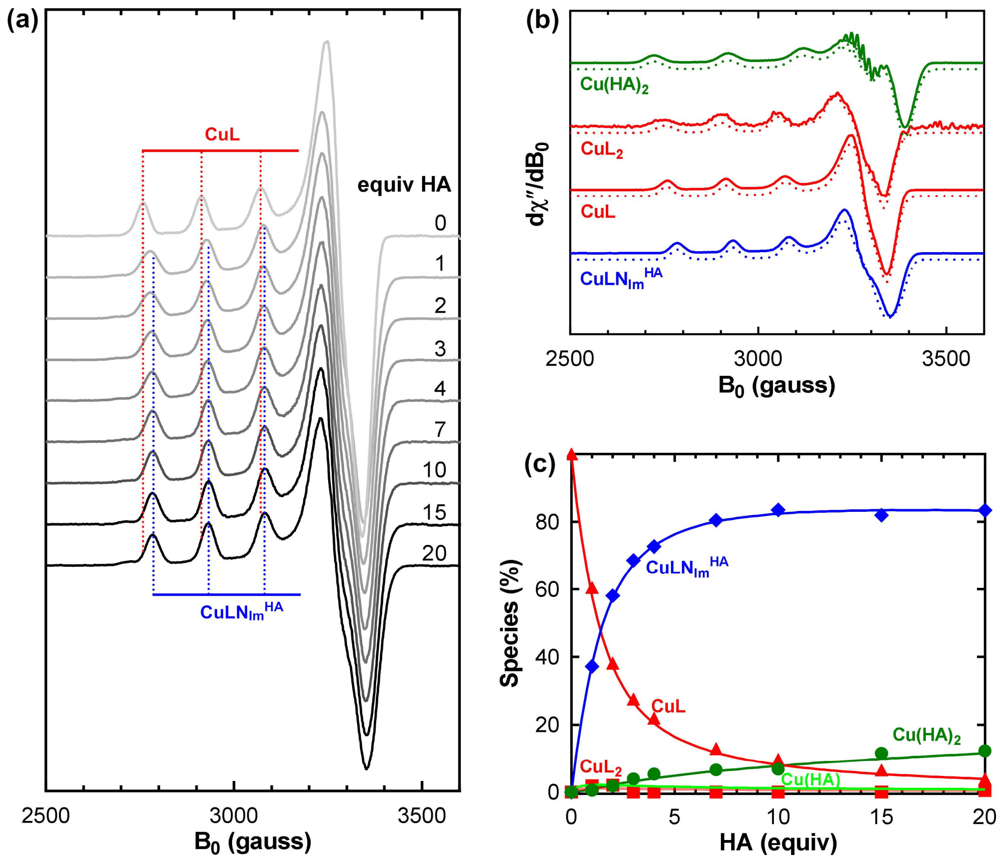

Figure S15. Determination of the stability of the ternary CuLN$_{Im}^{X}$ complex formed by X = histamine (HA) and the non-chlorinated PBT2 homologue (L). (a) CW-EPR spectra of ^{65}Cu/L/HA 1:1:*n* (*n* = 0–15) at 77 K in PBS pH 6.9 (0.225 mM L), originally reported by Kenche *et al.* [6]. **(b)** Normalized basis set used for the decomposition of the spectra in panel *a*. The spectrum of Cu(HA)$_2$ was obtained using a Cu/HA ratio of 1:50, the spectra of CuL and CuL$_2$ were obtained as described by Kenche *et al.* [6], and the spectrum of CuLN$_{Im}^{HA}$ was obtained as shown in Figure S16. The spectrum of Cu(HA) was not fitted but accounted for no more than 2% of the species (panel *c*). Dotted lines show spectra simulated using the parameters in Table S1. Spectra are drawn on a different scale from those in panel *a*. **(c)** Experimental species distributions (points) resulting from spectral decomposition (Figure S17) and theoretical distributions (lines) calculated using the relevant formation constants in Table S2. Experimental conditions: temperature, 77 K; microwave power, 10 mW; microwave frequency, 9.450 GHz; modulation amplitude, 8 G; sweep time, 180 s; time constant, 328 ms; averages, 4.

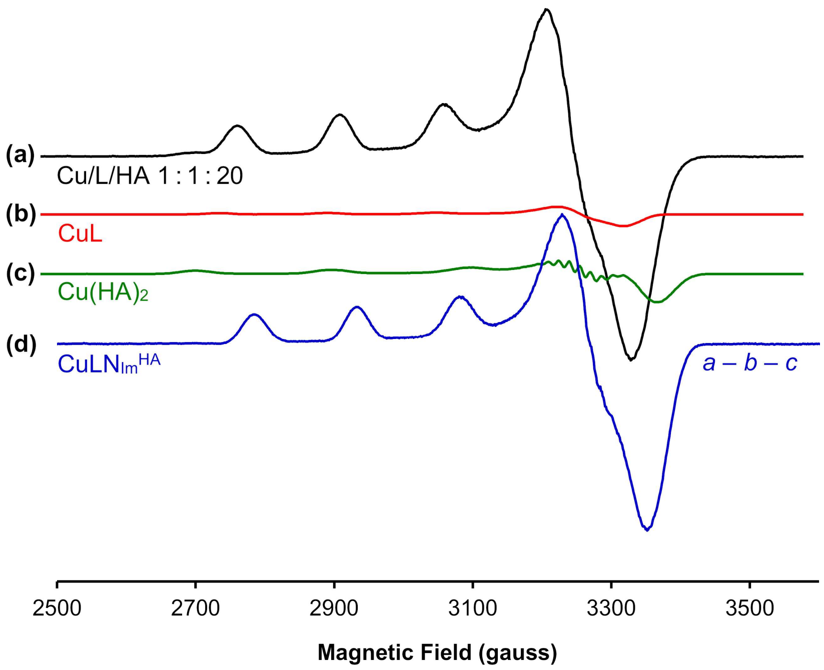

Figure S16. Isolation of the frozen-solution (77 K) X-band (9.450 GHz) EPR spectrum of $CuLN_{Im}^{HA}$ for the non-chlorinated PBT2 homologue (L). A mixture of Cu/L/HA 1:1:20 yields spectrum **(a)**. Using the species distributions (Figure S15c) resulting from the conditional binding constants for ${}^{c}K_{CuL}^{Cu}$, ${}^{c}K_{CuL_2}^{CuL}$, ${}^{c}K_{CuLN_{Im}^{HA}}^{CuL}$, ${}^{c}K_{Cu(HA)}^{Cu}$, and ${}^{c}K_{Cu(HA)_2}^{Cu(HA)}$ (Table S2), subtraction of **(b)** ≈4% CuL and **(c)** ≈12% $Cu(HA)_2$ yields a unique spectrum corresponding to **(d)** $CuLN_{Im}^{HA}$. At the above stoichiometry, the percentages of CuL_2 and are approximately zero; therefore, the spectrum is dominated by $CuLN_{Im}^{HA}$ and thus easily distinguished from that of all other species (Table S1, Figure S15b).

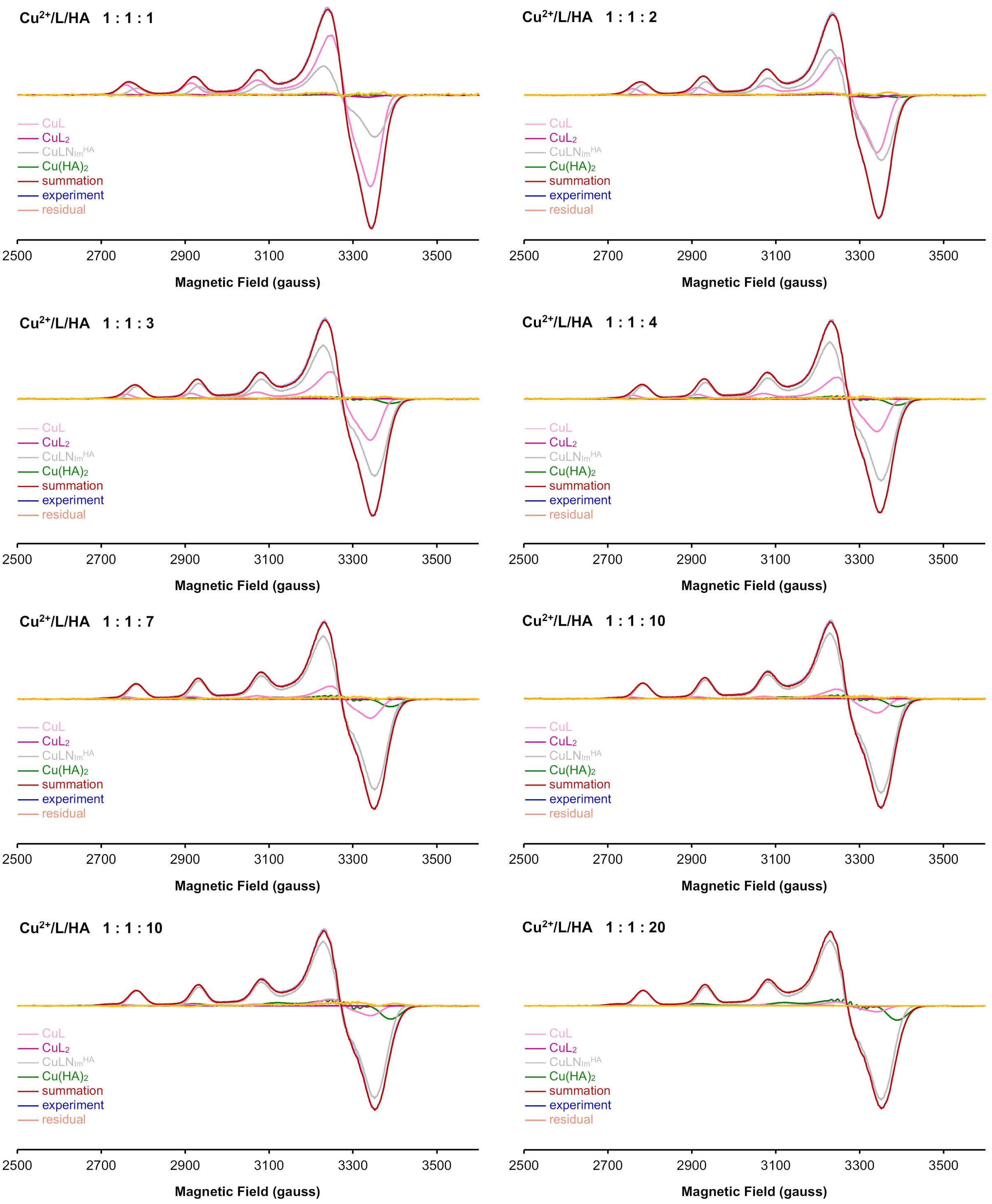

Figure S17. Decomposition of the frozen-solution (77 K) X-band (9.450 GHz) EPR spectra of Cu/L/HA 1 : 1 : *n* (*n* = 1–20) for the non-chlorinated PBT2 homologue (L). The corresponding percentages of each species are shown in Figure S15c. *Blue spectrum*, experiment; *pink spectrum*, CuL; *violet spectrum*, CuL_2; *grey spectrum*, $CuLN_{Im}^{HA}$; *green spectrum*, $Cu(HA)_2$; *red spectrum*, summation of CuL, CuL_2, $CuLN_{Im}^{HA}$, and $Cu(HA)_2$ spectra; *orange spectrum*, difference between the experimental spectrum (blue) and the summation (red).
